# A Comparative Analysis of Punicalagin Interaction with PDIA1 and PDIA3 by Biochemical and Computational Approaches

**DOI:** 10.3390/biomedicines9111533

**Published:** 2021-10-25

**Authors:** Giuliano Paglia, Lorenzo Antonini, Laura Cervoni, Rino Ragno, Manuela Sabatino, Marco Minacori, Elisabetta Rubini, Fabio Altieri

**Affiliations:** 1 Department of Biochemical Sciences “Alessandro Rossi Fanelli”, Sapienza University of Rome, Piazzale Aldo Moro 5, 00185 Rome, Italy; giuliano.paglia@uniroma1.it (G.P.); laura.cervoni@uniroma1.it (L.C.); marco.minacori@uniroma1.it (M.M.); elisabetta.rubini@uniroma1.it (E.R.); 2 Rome Center for Molecular Design, Department of Drug Chemistry and Technology, Sapienza University of Rome, Piazzale Aldo Moro 5, 00185 Rome, Italy; lorenzo.antonini@uniroma1.it (L.A.); rino.ragno@uniroma1.it (R.R.); manuela.sabatino@uniroma1.it (M.S.); 3 Enrico ed Enrica Sovena Foundation, 00199 Rome, Italy

**Keywords:** protein disulfide isomerase, PDIA3/ERp57, inhibitor, punicalagin, molecular docking, molecular dynamics, redox activity

## Abstract

In a previous work, it was shown that punicalagin, an active ingredient of pomegranate, is able to bind to PDIA3 and inhibit its disulfide reductase activity. Here we provide evidence that punicalagin can also bind to PDIA1, the main expressed form of protein disulfide isomerase (PDI). In this comparative study, the affinity and the effect of punicalagin binding on each protein were evaluated, and a computational approach was used to identify putative binding sites. Punicalagin binds to either PDIA1 or PDIA3 with a similar affinity, but the inhibition efficacy on protein reductase activity is higher for PDIA3. Additionally, punicalagin differently affects the thermal denaturation profile of both proteins. Molecular docking and molecular dynamics simulations led to propose a punicalagin binding mode on PDIA1 and PDIA3, identifying the binding sites at the redox domains *a’* in two different pockets, suggesting different effects of punicalagin on proteins’ structure. This study provides insights to develop punicalagin-based ligands, to set up a rational design for PDIA3 selective inhibitors, and to dissect the molecular determinant to modulate the protein activity.

## 1. Introduction

Protein disulfide isomerase A3 (PDIA3) is a multifunctional protein acting in multiple cellular compartments [1]. PDIA3 catalyzes proper disulfide bonds and thiol groups formation on target proteins through its thiol-oxidoreductase and disulfide isomerase properties [2].

PDIA3 is a 505 amino acids protein folded in four globular domains, named, respectively, *a*, *b*, *b’,* and *a’*, which were experimentally observed to be organized in a U-shaped scaffold (PDB ID: 3F8U) [3]. Even though each domain is characterized by a thioredoxin-like structure, only the first and fourth domains, *a* and *a’*, were reported as catalytically active, while the central domains provide binding sites for PDIA3 interactors [4,5].

Recently, increased knowledge on the PDIA3 system is being gradually added to shed light on its role in different cellular functions and human pathologies. There are several milestones in the functions of PDIA3. In the endoplasmic reticulum (ER), PDIA3 modulates the synthesis of newly synthesized *N*-glycosylated proteins interacting with lectin-binding proteins such as calreticulin and calnexin, as described [6]. In ER, PDIA3 regulates the redox status of major histocompatibility complex (MHC) class I during antigen presentation [7]. Moreover, PDIA3 has been proven as a modulator of the 1,25-dihydroxycholecalciferol (1α,25-(OH)_2_ or D3 vitamin) non-genomic response in cell membranes [8], or in the nucleus as an accessory protein to regulate the transcription factors redox state, i.e., STAT3 [9]. Recently, PDIA3 activity has been correlated with influenza A virus (IAV) replication mechanism. PDIA3 is upregulated in IAV-infected mice playing a key role in the folding of IAV-hemagglutinin [10]. In human hepatocellular carcinoma, PDIA3 downregulation inhibits cell proliferation and, through STAT3 signaling, induces apoptosis in agreement with the observation that PDIA3 knockdown reduces phosphorylated STAT3 and downstream STAT3-related protein levels [11]. PDIA3 has also been associated with human ovarian cancer chemoresistance. Co-treatment of PDIA3-siRNA and paclitaxel inhibits the STAT3 signaling pathway [12]. PDIA3 downregulation inhibits proliferation and increases apoptosis in acute myeloid leukemia [13], glioma [14], and colorectal cancer cells [15].

Despite the increasing number of studies, there is not yet available any chemical tool (inhibitor or activator) to further elucidate the PDIA3 role in the above-mentioned molecular mechanisms. Therefore, the identification of a selective PDIA3 ligand represents an important issue.

**Figure 1:**
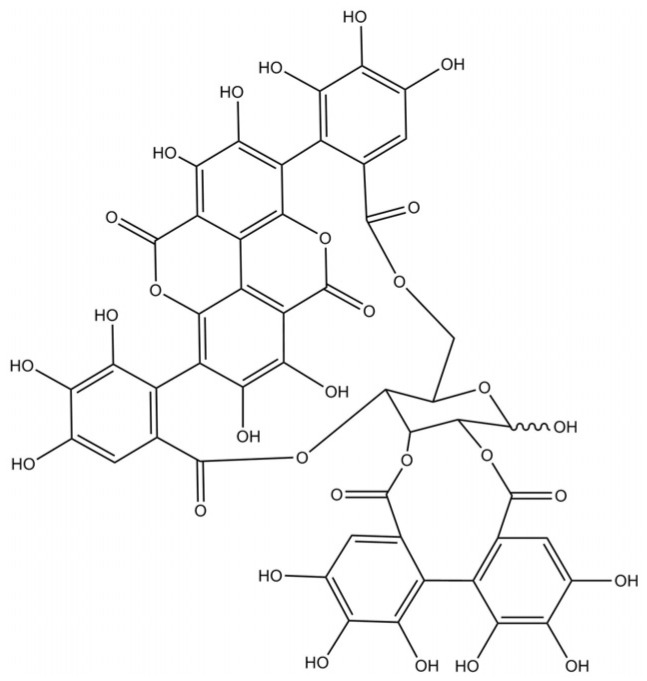
Molecular structure of punicalagin.

In a previous study [16], punicalagin was reported to bind to PDIA3 and to inhibit the reductase activity. Punicalagin (Figure 1) is a bioactive natural compound extracted from pomegranate fruit (*Punica granatum*) [17], in *Terminalia catappa* [18], *Terminalia myriocarpa* [19], and in *Combretum mole* [20]. It is an ellagitannin belonging to the polyphenol family, and it is soluble in water, unlike many other polyphenols. Punicalagin holds a wide range of pharmacological effects such as anti-inflammatory, hepatoprotective, antioxidant, and anti-cancer effects [21,22]. To investigate the punicalagin specificity in PDIA3 binding among PDI family members, a comparative study between PDIA3 and PDIA1 was performed. PDIA1 is the archetype of the PDI family, is the most abundant PDI in the endoplasmic reticulum [23], and shares a considerable similarity in structure and enzymatic functions with PDIA3 with respect to other PDI family members [24].

Biochemical studies have been carried out in order to compare PDIA3 and PDIA1 interactions and inhibitory effects of punicalagin. Subsequently, molecular dynamics and molecular docking studies have been performed as complementary techniques. Moreover, deep analyses on PDIAs-punicalagin interactions provide insights to set up a rational design for PDIA3 selective inhibitors and to dissect the molecular determinant to modulate the protein activity.

## 2. Materials and Methods

### 2.1. Chemicals

Reagents used in this study were purchased from Sigma-Aldrich (Milan, Italy) unless otherwise stated. EDTA (ethylenediamine tetra-acetic acid) 0.5 M solution pH 8.0 was purchased from IBI Scientific (Milan, Italy), Tris(hydroxymethyl)aminomethane for buffer solutions from Merck Millipore (Milan, Italy).

### 2.2. Recombinant Proteins: Production and Purification

Mature human recombinant PDIA3 protein was expressed and purified according to the procedure described by Trnkova et al. [25]. PDIA3 concentration was spectrophotometrically calculated by means of the extinction coefficient (ε_280_ in reduced form, 44,810 M^−1^cm^−1^).

The pOLR130 plasmid encoding mature human PDIA1 with an N-terminal His6x-tag [26] was generously provided by Dr. Lloyd Ruddock (University of Oulu, Finland). *E. coli* cells (strain BL21) were transformed with a vector containing the human PDIA1 sequence. After IPTG induction, cells were harvested and lysed. PDIA1 was purified by ammonium sulfate fractionation (30% to 75% saturation), nickel chromatography (Protino Ni-NTA column, Macherey-Nagel, Düren, Germany), followed by anion-exchange chromatography step (Macro-Prep Q column, BioRad, Milan, Italy). SDS-PAGE and Coomassie blue staining were performed to evaluate the protein purification, and concentration was spectrophotometrically estimated using the extinction coefficient (ε_280_ in reduced form, 45,567 M^−1^cm^−1^).

### 2.3. Measurements of Tryptophan Fluorescence Quenching

Protein-ligand interactions were evaluated according to the fluorescence quenching titration method using a SPEX-FluoroMax spectrofluorometer (Horiba Scientific, Piscataway, NJ, USA). Emission spectra were recorded from 300 to 400 nm with an excitation wavelength set at 290 nm. The emission slit width was fixed to provide a 2 nm bandpass for all experiments. Scan speed was set at 120 nm min^−1^. A total of 50 µM PDIA1 and PDIA3 stock solutions were reduced by adding DTT 5 mM. Aliquots of freshly reduced proteins were diluted (0.1 µM final concentration) in phosphate-buffered saline (PBS) pH 7.4 containing 0.2 mM EDTA and 0.1 mM DTT in a 10 mm path length quartz fluorescence cuvette under continuous stirring. The titrations were performed by stepwise additions, at 5 min time intervals, of punicalagin solution from 0 to 4 µM (punicalagin 0.2 mM in PBS pH 7.4 freshly prepared from a 5 mM stock solution in water). Blank spectra for each experiment (no protein added) were performed in parallel for background determination. The emission spectrum of the protein was recorded three times, and the average reading values were then used for quenching analyses as already described [27].

### 2.4. Isothermal Titration Calorimetry

Punicalagin interaction with PDIA3 and PDIA1 was investigated using isothermal titration calorimetry (ITC) with a MicroCal ITC200 (Malvern Instruments, Malvern, UK). Aliquots of freshly reduced protein were diluted (25 µM final concentration) in 20 mM Tris-HCl pH 8.0, 150 mM NaCl and 0.5 mM DTT. Protein solution was loaded in the sample cell (0.2 mL), and the syringe was filled with punicalagin 250 µM dissolved in the same buffer. A total of 20 ligand aliquots (the first aliquot of 0.4 µL in 0.8 s and the other 19 aliquots of 2 µL in 4 s) were gradually added to the sample cell at intervals of 200 s each other to allow baseline recovery. The syringe stirring speed was set at 800 rpm. Protein and punicalagin solutions were thoroughly degassed on the MicroCal’s Thermovac machine before loading to avoid air bubble formation during the titration. Data analyses were performed by Origin software version 8.0 (OriginLab, Northampton, MA, USA). The heat of dilution for the ligand was determined in parallel, and this value was subtracted from the integrated data before curve fitting. The integration of the area under each heat burst curve was determined. All injection heats as a function of the molar ratio were plotted and analyzed using the one set of sites model to provide the dissociation constants.

### 2.5. Differential Scanning Calorimetry

Protein stability in solution was assayed by differential scanning calorimetry (DSC) with a Microcal VP-DSC (Microcal, Northampton, MA, USA) testing both proteins. A 5 µM protein solution (from a 50 µM stock solution freshly reduced by adding DTT 5 mM) was prepared in 20 mM Tris-HCl pH 8.0, 150 mM NaCl, containing 0.2 mM EDTA, degassed at 25 °C and filled in the measurement cell. The reference cell was filled with protein-free buffer. A heating rate of 60 °C h^−1^ was set, and the measurement cell worked under an excess pressure of 0.2 kPa to avoid air bubble formation during the heating. The heat absorption during the denaturation process was measured by subtracting the instrumental baseline from the sample trace and normalizing for protein concentration. To test the effect of the ligand on protein stability, analysis was also performed on both proteins in the presence of punicalagin (25 µM final concentration). The melting points (T_m_, temperature at which excess heat capacity reaches a maximum) were directly observable from the thermogram. Nevertheless, data deconvolution using the Origin software provided by MicroCal was necessary to obtain T_m_ values.

### 2.6. Measurement of PDIAs Disulfide Reductase Activity

PDIAs disulfide reductase activity was monitored by using di-eosin glutathione disulfide (di-E-GSSG) as a fluorogenic substrate of PDIAs. Di-E-GSSG was synthesized by the reaction of eosin isothiocyanate with oxidized glutathione (GSSG) according to the method of Raturi and Mutus [28] with some modifications [29]. Protein activity was evaluated by monitoring the emission fluorescence increase (λ_em_ = 545 nm and λ_ex_ = 525 nm), and the effect of punicalagin (concentration ranging from 0.2 to 50 µM) was tested. Inhibition constants were extrapolated by GraphPad Prism 8.0 software (GraphPad Software, San Diego, CA, USA), plotting the obtained data as logarithm dose-response curves.

## 3. Results

### 3.1. Biochemical Studies

#### 3.1.1. Assessment of PDIA-Punicalagin Interactions by Intrinsic Fluorescence Spectroscopy

**Figure 2:**
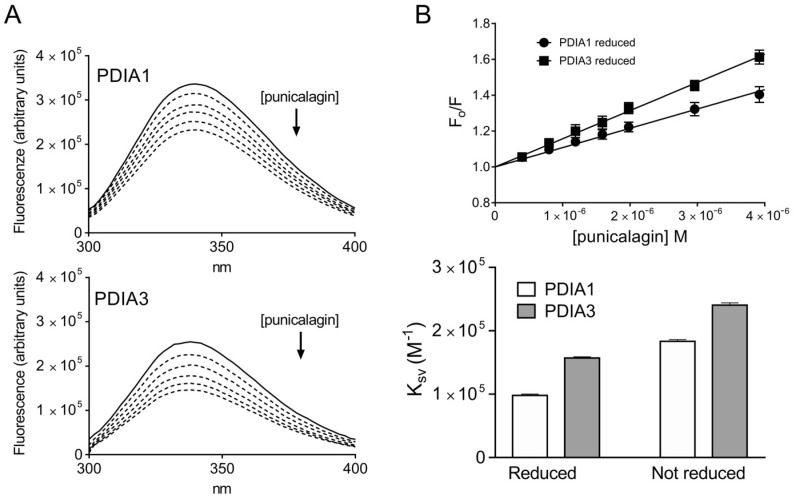
Protein fluorescence quenching analysis of PDIA1 and PDIA3 in the presence of punicalagin: (**a**) Fluorescence quenching spectra of reduced PDIA1 and PDIA3 alone (solid line) and after stepwise addition of punicalagin (dotted line) (pH 7.4, 25 °C, and λ_ex_ = 290 nm, (PDI) = 0.1 × 10^−6^ M, (punicalagin) = 4 × 10^−6^ M final concentration); (**b**) Stern–Volmer plot of quenching data of reduced PDIs in the presence of increasing concentrations of punicalagin. Stern–Volmer constants (K_SV_) calculated for the different systems tested. Data represent the mean of at least three independent experiments, and error bars indicate SEM.

Quenching of intrinsic fluorescence of both PDIAs upon ligand binding was evaluated. Each protein possesses an intrinsic fluorescence given by aromatic amino acids content, and the tryptophan residues are the dominant source [30]. PDIA3 contains three tryptophan residues, two of them, W56 and W405, are located next to the active sites, respectively, in the *a* (C57-C60) and *a’* (C406-C409) domains, whereas the third tryptophan, W279, is on the *b’* domain and is only partially exposed to the solvent [29]. Instead, PDIA1 contains five tryptophan residues, W52, W128 sited on *a* domain, W364, W396, W407 on the *a’* domain. W52 and W396 residues, homologous to W56 and W405 PDIA3 residues, are located next to the redox sites (C53-C56 and C397-C400) and are mostly exposed to the solvent. Considering PDIA1 3D structure, W407 residue appears buried, while W128 and W364 residues are partially and fully exposed, respectively. Upon ligand binding, the fluorescence intensity of both proteins could be quenched, suggesting that an interaction protein-ligand nearby tryptophan residues is taking place (Figure 2A).

Quenching of intrinsic tryptophan fluorescence at 338 nm has been analyzed by Stern–Volmer equation (F_o_/F = 1 + K_SV_[L]), where F and F_0_ are the fluorescence intensity, respectively with or without ligand (L), and K_SV_ is the Stern–Volmer constant value. Based on obtained results, punicalagin is able to quench the fluorescence of both proteins with a Stern–Volmer constant always greater than 1.0 × 10^4^ M^−1^ (Figure 2B and Table 1). Considering that the Stern–Volmer constant is equal to the quenching constant multiplied by the average lifetime of the fluorophore (K_SV_ = K_q_τ), where the tau value for tryptophan is in the order of 1.0 × 10^−8^ s [31,32], K_q_ values are always greater than 1.0 × 10^10^ M^1^s^−1^, the maximum quenching rate for diffusion collision, suggesting that a static interaction occurs [33]. In order to further characterize the binding process, data were analyzed using the equation described by Bi et al. [34] and the reiterative calculation process described by Sun et al. [35], providing an estimation of the K_d_ values (Table 1). Punicalagin binds both PDIAs in the micro-molar order of magnitude, showing a slightly greater affinity toward PDIA3, which tryptophan residues appear more quenchable upon punicalagin binding. In addition, in both proteins, the intrinsic fluorescence analyzed in reducing conditions appear less affected by punicalagin, suggesting either a minor affinity or less exposure of tryptophan residues for the protein in the reduced conformation.

#### 3.1.2. Analysis of PDIA-Punicalagin Interactions by Isothermal Titration Calorimetry

To a deeper characterization of PDIA-punicalagin interaction, ITC was used as it depicts the standard way to study protein-ligand interaction providing detailed thermodynamic parameters (entropy change ΔS, enthalpy change ΔH, binding constant) in solution. The enthalpy change is generally defined as the energy change resulting from all non-covalent protein-ligand interactions. Instead, the entropy change is given by the solvent entropy change upon ligand binding, as well as conformational entropy change and loss of rotational and translational degrees of freedom upon complex formation [36].

Considering the difficulty of assessing a fully oxidized state, proteins were analyzed in the presence of reducing agents after being freshly reduced. Obtained data confirmed the protein-ligand complex formation in both proteins, with an estimated K_d_ of 1.0 µM and 1.2 µM for PDIA1 and PDIA3, respectively (Table 2). K_d_ constant values were quite similar for the two proteins and slightly lower compared to the dissociation constants measured by tryptophan fluorescence quenching analysis. This may suggest that, although both proteins seem to bind punicalagin with a comparable affinity, the binding could differently involve tryptophan residues and influence protein intrinsic fluorescence. Both protein-punicalagin interactions are characterized by negative enthalpy change and positive entropy change (Table 2). Both parameters positively contribute to reducing the Gibbs free energy. Nevertheless, TΔS is much greater than the ΔH factor, meaning that the entropic factor represents the driving force of both interactions. The negative ΔH suggests that non-covalent interactions at the binding surface occur, whereas positive TΔS indicates that the rearrangement of water molecules, upon ligand binding, plays a key role by increasing the solvent degrees of freedom. However, the thermodynamic parameters obtained from ITC data seem to be different for PDIA1 and PDIA3, suggesting a slightly different interaction with punicalagin.

#### 3.1.3. Thermally Induced Transitions of PDIAs upon Punicalagin Binding

**Figure 3:**
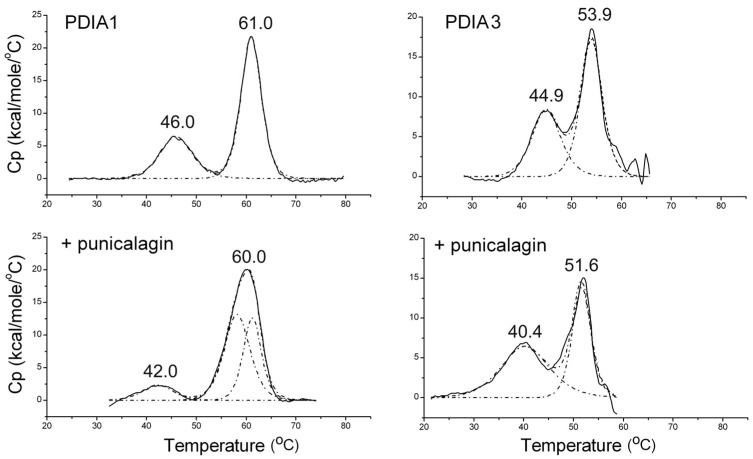
Thermal denaturation profiles of reduced PDIA1 and PDIA3 obtained by DSC. Proteins, 5 µM concentration, were analyzed in the absence and presence of 25 µM punicalagin. The transition temperature (T_m_) of each endothermic peak is reported. The dotted lines show the best fit obtained by deconvolution of each thermogram in non-two-state transitions.

Any conformational change induced by punicalagin binding may affect the stability of PDIA3 and PDIA1 proteins, and this was therefore evaluated by means of differential scanning calorimetry (DSC). First, the thermally induced denaturation process of both PDIAs in the reduced form was assessed. The thermal profile of both proteins displayed two endothermic peaks (Figure 3), which may represent different denaturation steps in which, respectively, partial and complete protein denaturation occurs. Deconvolution of data suggests that proteins underwent a two-step denaturation process (non-two-state transition). Despite the PDIA3 and PDIA1 sharing a suitable structure similarity, PDIA3 showed lower stability with the transition temperature (T_m_) of the second step at 53.9 °C, with respect to 61.0 °C of PDIA1. Instead, the temperature difference between the first two peaks is less marked, with a T_m_ = 44.9 °C for PDIA3 with respect to 46.0 °C of PDIA1 (Figure 3). As reported, switching from an open to a closed conformation occurs in PDIA1, with a greater closeness between *a* and *a’* domains in the reduced form [37]. It can be hypothesized that the first thermal transition could be the result of a partial protein unfolding leading to an open conformation similar to that adopted in the oxidized form.

Tryptophan fluorescence quenching results did not show a shift in the intrinsic fluorescence emission upon punicalagin binding. Therefore, it could be deduced that no noticeable conformational change occurs directly upon punicalagin binding. However, punicalagin binding influenced the denaturation profile of both proteins, negatively affecting PDIA3 stability. In fact, the T_m_ of the two endothermic peaks of PDIA3 decreases from 44.9 to 40.4 °C and from 53.9 to 51.6 °C, respectively. In the case of PDIA1, T_m_ values of the second peak were quite similar, 61 and 60 °C, while T_m_ of the first peak decreased from 46 to 42 °C. In both cases, the first thermal transition was more influenced by punicalagin binding, but only in PDIA1 did the presence of punicalagin greatly reduce the peak area associated with the first transition, corresponding to the enthalpy variation (ΔH), and broadened the second endothermic peak (Figure 3). This suggested that the binding of punicalagin can affect the stability of the two proteins differently.

#### 3.1.4. Punicalagin Effect on PDIA3 and PDIA1 Reductase Activity

**Figure 4:**
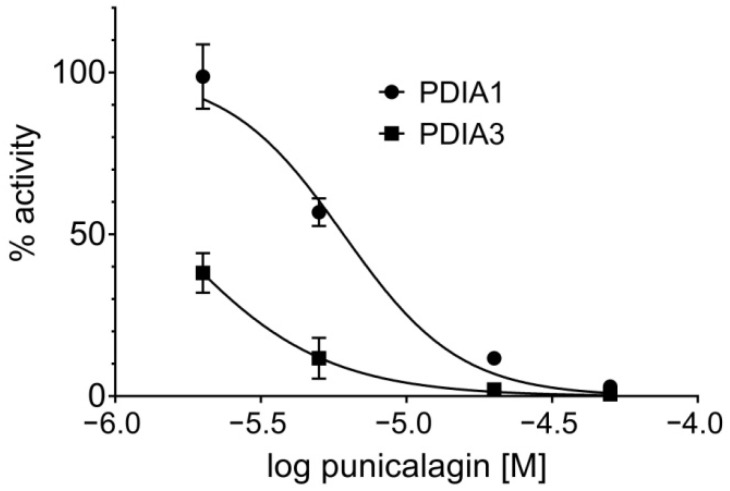
Dose-response curve of punicalagin showing the inhibition of PDIA1 and PDIA3 reductase activity using di-E-GSSG as substrate. Each point represents the average of at least three independent measurements. PDIA1, IC_50_ = 6.1 µM, 95% confidence from 5.5 to 6.7 µM; PDIA3, IC_50_ = 1.5 µM, 95% confidence from 1.3 to 1.6 µM).

Punicalagin effects have also been studied on the enzymatic activity of PDIA3 and PDIA1. It is well known that both proteins catalyze oxidoreductase and isomerase reactions involving thiol groups and disulfide bonds [2,38]. PDIs’ reductase activity was evaluated using di-E-GSSG as fluorogenic substrate and was calculated as the initial velocity in fluorescence increased due to the reduction in the fluorogenic substrate. The previous investigation identified punicalagin as an inhibitor of PDIA3 reductase activity [16], as a non-competitive inhibitor with a K_i_ of 0.39 µM, and this was herein confirmed. For comparison purposes, the punicalagin inhibitory effect was extended to PDIA1. Results were analyzed as logarithmic dose response, and the half-maximal inhibitory concentrations (IC_50_) were evaluated (Figure 4). Obtained data show that punicalagin has a better inhibitory effect toward PDIA3 (IC_50_ 1.5 ± 0.2 µM) compared to PDIA1 (IC_50_ 6.1 ± 0.6 µM).

### 3.2. Computational Studies

**Figure 5:**
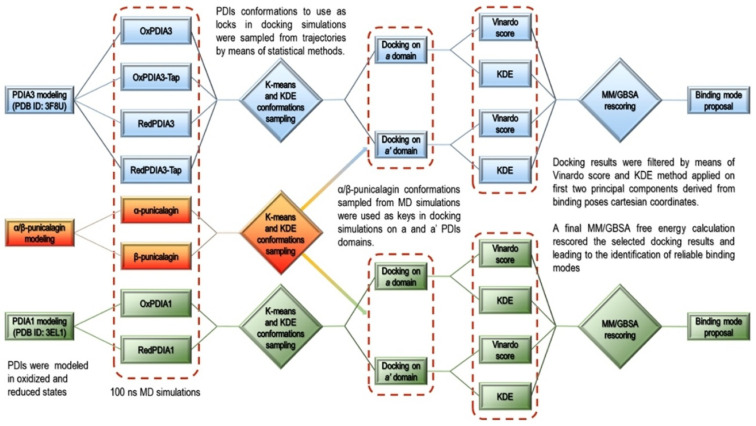
Scheme of the computational chemistry procedure conducted to provide a putative binding mode for punicalagin on PDIA3 and PDIA1. See text for details.

To rationalize the herein presented experimental data and provide the putative binding mode for punicalagin on PDIA3 and PDIA1, a computational chemistry procedure was conducted combining molecular dynamics (MD) simulations with molecular docking simulations as summarized in the scheme depicted in Figure 5:

1. Modeling of the PDIAs crystal structures in oxidized and reduced states;
2. MD simulations of the modeled PDIAs;
3. Analysis of the MD trajectories and conformations’ sampling for the subsequent ensemble molecular docking (cross-docking) [39] simulations;
4. α and β-punicalagin MD-based conformational analysis for the molecular docking into *a* and *a’* domains of PDIAs sampled conformations;
5. Analysis of the molecular docking results by means of statistical techniques and docking score ranking allowed binding poses selection for final rescoring with MM/GBSA [40].

#### 3.2.1. Molecular Dynamics Simulations

As no experimental PDIA3-ligand complex is yet available, MD simulations were run to sample PDIA3 conformational flexibility to overcome the lack of ligand-protein induced-fit sampling frames to be used in molecular docking simulations to obtain insight on the putative punicalagin binding mode [41]. Starting from the PDIA3 crystal structure (PDB ID:3F8U) [3], the molecular systems were built and then subjected to MD simulation following the procedures reported in the “*Computational Methods*” section (Supplementary Material Sections S1.1 and S1.2). The PDIA3 and PDIA3-Tap molecular systems were modeled in both the reduced (Red) and oxidized (Ox) forms for either apoproteins or complexed with tapasin (PDIA3_Ox_, PDIA3_Ox_-Tap, PDIA3_Red_, and PDIA3_Red_-Tap). A total of 400 ns MD simulation trajectories were firstly analyzed, collecting the protein backbone root mean square deviation (RMSD) to investigate protein stability. Tapasin-containing complexes (PDIA3_Ox_-Tap and PDIA3_Red_-Tap) showed a stable RMSD profile with values ranging from 3 to 5 Å, while the free PDIA3_Ox_ and PDIA3_Red_ systems returned an increasing trend up to 12–14 Å (Figure S1A). This difference was also consistent with the RMSD probability density function plot (Figure S1B), likely due to the domains’ high mobility. In fact, during the PDIA3s’ MD simulations, the proteins were free to move, while in the complexed systems, tapasin induced some structural constraints on both *a* and *a’* domains resulting in lower RMSD values. As punicalagin is also a PDIA1 inhibitor, the calculations were replicated on the PDIA1 system. To this, the PDIA1 starting crystal structure (PDB ID:4EL1) [37] was prepared as described in the “*Computational Methods*” section (see Supplementary Material Sections S1.1 and S1.2), solvated, and subjected to MD simulation for either oxidized (PDIA1_Ox_) or reduced state (PDIA1_Red_). Although experimentally available (PDB ID:4EKZ) [37], for consistency with the PDIA3 system, the PDIA1 reduced state was modeled starting from the oxidized PDIA1 crystal. The RMSD between the reduced PDIA1 crystal structure (PDB ID: 4EKZ) and the modeled one after the initial MD equilibration was 4.03 Å, while between the oxidized (PDB ID: 4EL1) and the reduced (PDB ID: 4EKZ) PDIA1 crystal structures was 6.87 Å. The RMSD fluctuations range (12–13 Å) and its trend observed from the MD trajectory analysis overlapped those observed for PDIA3_Ox_ and PDIA3_Red_ (Figure S1). Notably, PDIA1_Red_ reached the equilibrium after 10 ns at an RMSD value of ~11 Å. Upon deeper analysis, the distance between *a* and *a’* domains along the PDIA1 simulations was collected (Figure S4) and revealed PDIA1_Red_ switching from an open to a closed conformation during the first 10 ns (Figure S5). The latter agreed with experimental data showing a greater closeness between PDIA1 *a* and *a’* domains in the reduced form [37]. Further analysis on PDIA3 and PDIA1 trajectories are reported in Supplementary Material Section S2.1.

#### 3.2.2. Molecular Docking Simulations

As introduced above, PDIAs trajectories were analyzed, and a series of snapshots (60 for PDIA3 and 30 for PDIA1) were sampled (see Supplementary Material Sections S1.3 and S2.2) to run molecular docking simulations. As α and β-punicalagin (Scheme S1) are characterized by a cyclized, highly constrained chemical structure and considering the smina limitations (the cycles are treated rigidly), molecular docking simulations were conducted using a rigid body docking procedure. To fulfill the lack of conformational flexibility, MD simulations of α and β-punicalagin were carried out to sample punicalagin conformations (details in Supplementary Material Sections S1.4 and S2.3).

**Figure 6:**
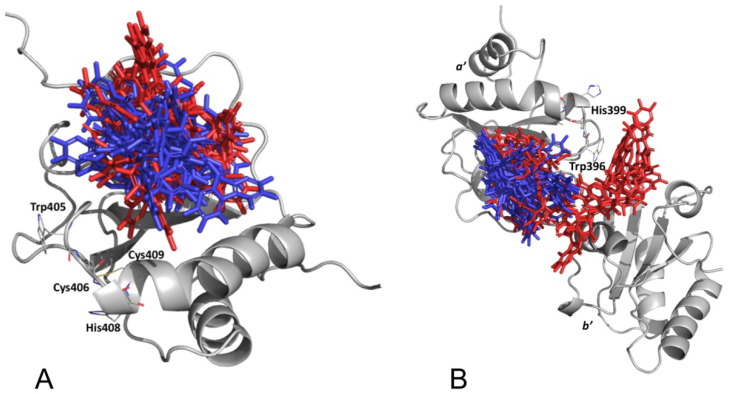
Selected α and β-punicalagin docking results for PDIA 3 (**A**) and PDIA1 (**B**) *a’* domain: conformation selected by Vinardo score are depicted in red, while those selected by KDE are blue.

The selected α and β-punicalagin conformations (40 for each) were used as keys and docked onto the *a* and *a’* domains (locks) of PDIA3 sampled conformations. Among the resulting 24,000 docked poses for each domain, conformations were selected using either the highest Vinardo score (10 conformations) or a statistical approach applying kernel density estimation (KDE) on poses’ heavy atoms cartesian coordinates first two principal components (10 conformations). For PDIA3 *a* domain (Figure S11A), the α and β-punicalagin conformations selected using the two approaches displayed partial overlap, returning as the mean distance between poses’ center of mass (MDCOM) a value of 7.4 Å. For the PDIA3 *a’* domain (Figure 6A), the conformations sampled using the two approaches showed a very suitable overlap, being all the predicted binding modes in the same pocket occupying the same volume (MDCOM 2.9 Å), indicating a convergent way to select the docked α and β-punicalagin conformations. The 20 selected poses were used for free energy calculations for final rescoring and selecting the most likely binding conformation.

The same docking procedure was applied for PDIA1 *a* and *a’* domains of 30 selected MD snapshots, returning 12,000 binding poses for each PDIA1 catalytic domain. Again, the binding pose selection for free energy calculations was accomplished by means of the smina’s Vinardo score ranking and KDE algorithm. Selected docking results on PDIA1 *a* domain (Figure S14A) showed two distinct conformations clusters matching the two selection approaches (MDCOM 8.7 Å). Selected docking results on the *a’* domain (Figure 6B) were mainly grouped into a cluster occupying a domain hydrophobic pocket (MDCOM 7.4 Å), the same identified as punicalagin binding site on PDIA3 *a’* domain. However, few poses were located outside this cluster, at the *a’/b’* domains interface (Figure 6B and Figure S15).

#### 3.2.3. Punicalagin Binding Mode Selection by Free Energy Calculations

The selected α and β-punicalagin binding poses from molecular docking simulations into *a* and *a’* domains were merged with the respective locks obtaining a number of complexes to calculate the binding free energy (ΔG_bind_) by means of the MM/GBSA method.

**Figure 7:**
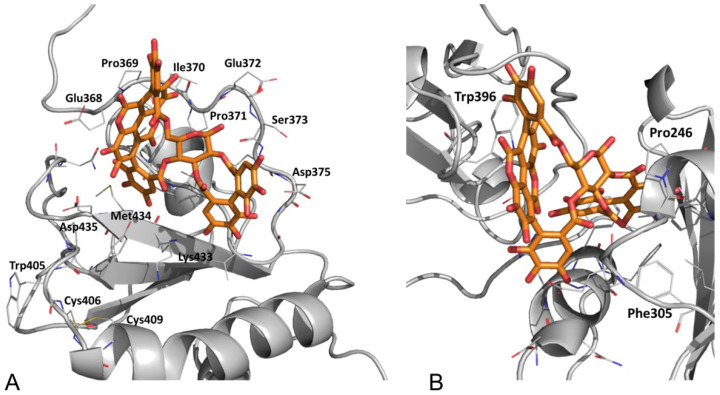
Top-ranked β-punicalagin binding modes on (**A**) PDIA3 *a’* domain (−39.2 kcal mol^−1^) and (**B**) PDIA1 *a’* domain (−35.2 kcal mol^−1^).

In agreement with experimental data [16], lower estimated ΔG_bind_ values were obtained for punicalagin complexed with PDIA3_Ox_ *a’* domain. The top-ranked calculated ΔG_bind_ for β-punicalagin on PDIA3 *a* domain was −39.2 kcal mol^−1^ while on PDIA3 *a’* domain was −49.9 kcal mol^−1^. In both domains, β-punicalagin showed a higher affinity with respect to the α epimer. The binding conformation for β-punicalagin was directly selected for the *a’* domain as the top-ranked one (Figure 7A), showing a lower ΔG_bind_ of about 8 kcal mol^−1^ than the α epimer. This result lets us uniquely define a reliable binding mode for β-punicalagin on PDIA3 *a’* domain where the ligand is settled in a hydrophobic pocket constituted by Val378, Val380, Val381, and Val382. Main polar interactions are with Val381, Lys433, Val378, Gly376, Asp435, Ser373 backbone atoms and with Asn439 side chain (Figure 7A).

Binding free energy calculations indicated PDIA1 *a’* as the punicalagin preferred binding domain and pointed β-punicalagin as the favored epimer for both catalytic domains. Differently for PDIA3, β-punicalagin affinity was calculated to be higher for PDIA1_Red_. The top-ranked calculated ΔG_bind_ for β-punicalagin on PDIA1 *a* domain was −42.7 kcal mol^−1^ while for the *a’* domain was −56.8 kcal mol^−1^. The top-ranked binding mode on the PDIA1 *a’* domain showed a ΔG_bind_ difference of about 11 kcal mol-1 with the second one. These results let to hypothesize a reliable binding mode for β-punicalagin on PDIA1_Red_ *a’* domain where the ligand is inserted in a hydrophobic pocket at the *a’/b’* interface, characteristic of the closed conformation, constituted by Pro246, Phe304, Phe305, Phe240, Phe349, Leu258, Ile318, Ile301, Arg300 and Trp396, which interacts with ellagic acid moiety trough a π–π stacking. Main polar contacts are established with Arg300, Asp297 (Figure 7B and Figure S17).

#### 3.2.4. Punicalagin Binding Mode on PDIA3 Refinement by MD Simulation

**Figure 8:**
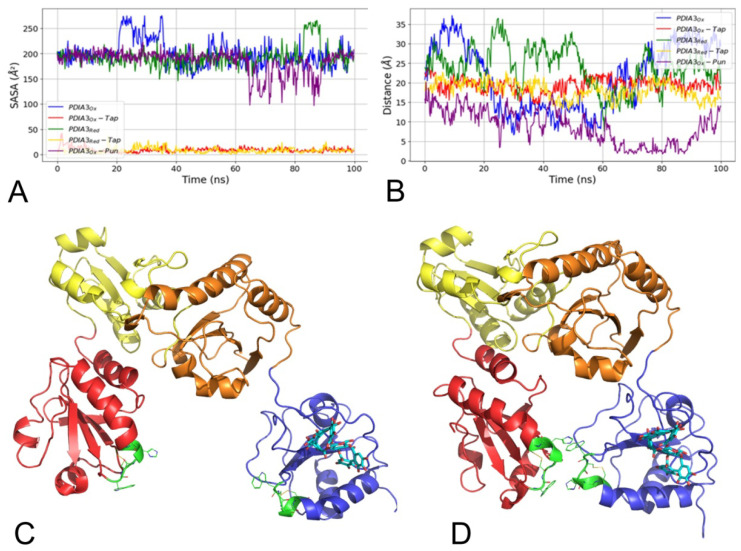
(**A**) Solvent accessible surface area (SASA) time series plot and (**B**) closest distance between *a* and *a’* domains over time plot calculated along MD simulations. (**C**) PDIA3_Ox_ in “open” and “closed” (**D**) conformation. PDIA3 *a* domain is depicted in red, *b* is yellow, *b’* is orange, *a’* is blue, WCGHC patterns are green lines while punicalagin is depicted as cyan sticks.

As the predicted β-punicalagin binding mode on PDIA3 was not found in close contact with Trp405 quenching residue [16], an investigation on how β-punicalagin could modulate PDIA3 disulfide reductase activity was conducted by means of a 100 ns MD simulation. Trajectory analysis then focused on Trp405 solvent accessible surface area (SASA) collection. The β-punicalagin/PDIA3_Ox_ complex (PDIA3_Ox_-Pun) showed a different Trp405 SASA trend with respect to the other trajectories (Figure 8A), showing a Trp405 burial in the second half of the simulation. A visual inspection indicated that β-punicalagin was not responsible for the PDIA3_Ox_-Pun SASA fluctuation trend. Nevertheless, *a* and *a’* domains were approaching each to the other to make contacts adopting a “closed” or “collapsed” conformation (Figure 8C,D) never observed during simulations of either PDIA3_Ox_ or PDIA3_Red_. Analysis of the distance fluctuations between the *a* and *a’* domains clearly showed the under-closing conformation of PDIA3_Ox_-Pun, reaching distance values less than 5 Å in the second half of the simulation (Figure 8B). RMSD analysis of PDIA3_Ox_-Pun (Figure S13A) returned a similar trend to PDIA3_Ox_ and PDIA3_Red_ while the root mean square fluctuation (RMSF) (Figure S13B) indicate reduced flexibility of PDIA3_Ox_-Pun complex, with lower RMSF values than PDIA3_Ox_ and PDIA3_Red_. These results could account in part for the quenching experiments and let to speculate on an inhibition mechanism based on the modulation of PDIA3 flexibility regulating the availability of the catalytic sites.

#### 3.2.5. PDIA3/PDIA1 β-Punicalagin Binding Mode Comparison

**Figure 9:**
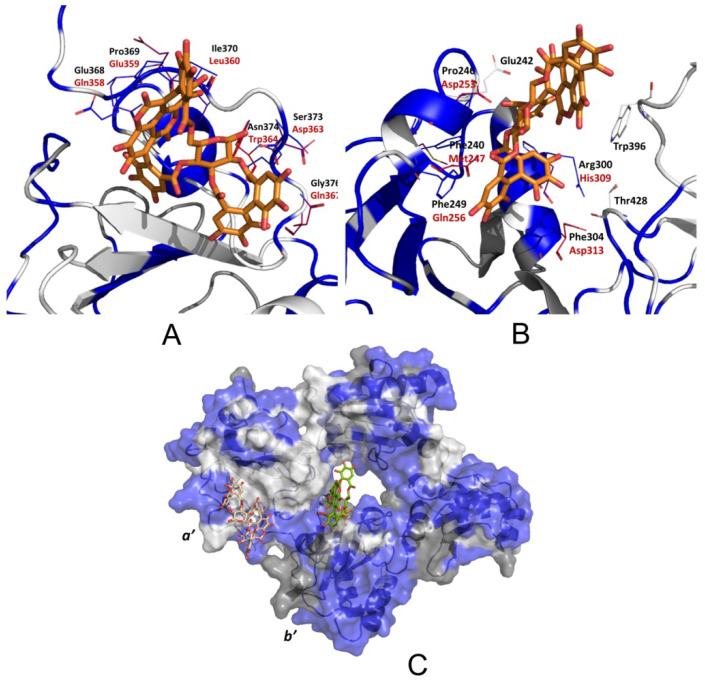
Comparative analysis of predicted β-punicalagin binding modes on PDIA3 and PDIA1. Residues are colored by conservation: white indicates conserved regions, blue nonconserved regions, and dark gray not aligned regions. (**A**) Predicted β-punicalagin binding mode on PDIA3 *a’* domain. Nonconserved residue names are reported in black for PDIA3 and red for PDIA1. PDIA1 nonconserved residues are depicted as lines and colored from blue to red according to their score in the alignment matrix. (**B**) Predicted β-punicalagin binding mode on PDIA1 *a’/b’* domains interface. Nonconserved residue names are reported in black for PDIA1 and in red for PDIA3. (**C**) Predicted β-punicalagin binding modes for PDIA3 (wheat) and PDIA1 (green) reported on the same structure (PDIA1) for comparison. Only *a’* and *b’* domains are visible.

Sequence identity between full-length PDIA3 and PDIA1 is 36%, while identity between PDIA3 and PDIA1 *a* domains is 50% and between PDIA3 and PDIA1 *a’* domains is 45%, being the *a’* the less conserved one. Sequence analysis was also used to visually inspect PDIs binding pocket conservation (Figure 9). Results on PDIA3 showed punicalagin settling on a conserved hydrophobic pocket approaching the 358–376 loop (Figure 9A). The latter section, corresponding to the *x* linker in PDIA1 [37], is also where the nonconserved interacting residues are located. Results on PDIA1 showed punicalagin binding into a hydrophobic pocket peculiar of the PDIA1_Red_ closed conformation (Figure 9B). Although a few residues are conserved (Glu242PDIA1/Glu249PDIA3, Thr428PDIA1/Thr437PDIA3 and Trp396PDIA1/Trp405PDIA3), this pocket is not present in PDIA3 and might be responsible for the punicalagin/PDIA1 main interactions (i.e., π–π stacking with the ellagic acid moiety). The proposed β-punicalagin binding modes on PDIA3 and PDIA1 are in two different binding pockets suggesting different effects of this molecule on proteins’ structure, in agreement with presented experimental data, as well as a different inhibition mechanism. In the case of PDIA3, β-punicalagin could modulate protein flexibility and consequently the equilibrium between the “open” and the “closed” conformations (as indicated by PDIA3_Ox_-Pun simulation), while PDIA1 could be targeted by β-punicalagin in the “closed” state exerting a stabilizing effect through binding in the above-described pocket.

Indeed, PDIA1 flexibility was an already discussed theme in scientific literature. PDIA1 has been subjected to redox-dependent structural changes and is characterized by high interdomain mobility [37,42,43]. The *b’xa’* domains are the most dynamic, and this is crucial to properly interact with substrates while exerting the chaperone function [44]. Reduced PDIA1 is more stable than the oxidized one and more resilient to thermal stress leading to hypothesize a rearrangement in a “closed” conformation in which the *x* linker plays a key role in the *a’* domain positioning [45]. These reports agree with the herein reported PDIA1_Red_ MD simulation (see Supplementary Material Section S2.1), in which PDIA1 adopted a “closed” conformation during the first 10 ns. Such conformation could not be able to properly interact with substrates, and β-punicalagin might stabilize it, avoiding PDIA1 opening. The binding mode proposed for PDIA3 presented β-punicalagin interacting with a hydrophobic pocket approaching the flexible loop connecting *a’* and *b’* domains, corresponding to PDIA1 *x* linker. This could explain the *a/a’* approach observed in PDIA3_Ox_-Pun MD simulation and then the inhibition mechanism.

## 4. Discussion

In a previous work, it was shown that punicalagin, found in forms α and β in pomegranates as well as other natural sources, was able to bind to PDIA3 and inhibited its disulfide reductase activity. Herein we provide evidence that punicalagin can also bind to PDIA1, the main expressed form of PDI. In this comparative study, the affinity and the effect of punicalagin binding on both proteins were evaluated, and computational studies were used to identify the putative PDIA1 and PDIA3 binding sites. Although the findings indicated punicalagin capable of binding both proteins with a similar affinity, further biochemical evidence revealed several differences due to the effect of binding. Punicalagin was able to better quench the fluorescence of PDIA3 and showed a higher inhibitory effect on protein reductase activity for PDIA3 than that on PDIA1. Additionally, punicalagin differently affects the thermal denaturation profile of both proteins. The decrease in Tm for PDIA3_Ox_-Pun complex suggested a preferential binding for the unfolded protein conformation, while PDIA1_Ox_-Pun complex was characterized by a marked reduction in the peak area associated with the first thermal transition (likely the “closed-open” conformational switch that the protein in the reduced form undergoes).

Computational investigations identified PDIA1 and PDIA3 *a’* redox domains as the most favorable punicalagin binding sites, which sequence and structure mostly differ between the two proteins. Furthermore, punicalagin binding sites were suggested to be in two different pockets on PDIA3 and PDA1, in agreement with the quenching and thermal denaturation profiles.

The proposed PDIA3-punicalagin binding was predicted to settle in a hydrophobic pocket on the *a’* domain, and the ligand showed a preferential binding for the oxidized form. MD simulations indicate reduced flexibility of the PDIA3_Ox_-Pun complex, modulating the availability of the catalytic sites. Although a direct interaction with tryptophan residues was not evident, these results could account in part for the higher intrinsic fluorescence quenching observed for PDIA3-punicalagin interaction and let to speculate on an inhibition mechanism based on the modulation of PDIA3 redox domain flexibility.

The binding mode proposed for PDIA1 showed punicalagin in a hydrophobic pocket at the *a’/b’* interface, experimentally observed in the closed conformation adopted by the protein in the reduced form (PDIA1_Red_). This agreed with the variation in the PDIA1 thermal profile induced by punicalagin. In fact, the area associated with the first endothermic peak, probably related to the closed-open transition, is greatly reduced while the second peak appears broadened. In this case, the inhibitory mechanism could be mainly based on the stabilization of the protein in the reduced form.

ITC thermodynamic data agreed with the proposed punicalagin binding modes. The PDIA1-punicalagin complex was characterized by a higher enthalpy contribution, likely resulting from several interactions stabilizing the binding (i.e., π–π stacking with the ellagic acid moiety). On the other hand, binding of punicalagin to PDIA3, as seen in PDIA3_Ox_-Pun MD simulation, could modify the protein flexibility and, therefore, solvent accessibility, thus explaining the higher entropic contribution in this interaction.

PDI family consists of a large number of versatile proteins that can catalyze the oxidation/reduction/exchange of disulfide bonds within substrates. PDIs are involved in a wide range of cellular functions and are differently associated with several pathological conditions, including neurodegeneration and cancer. Thus, the search for specific and selective inhibitors/modulators of their activity is important to allow an aimed therapy. Here is presented evidence that punicalagin, a naturally occurring molecule, is able to differently bind and affect the structure and activity of PDIA1 and PDIA3, two of the most abundant proteins of the family. Molecular docking and simulation analyses provided information on the different punicalagin binding modes on PDIA1 and PDIA3. These findings will be useful to develop punicalagin-based ligands and to set up a rational design for PDIs selective inhibitors.

## Figures and Tables

**Table 1:** Stern–Volmer constants (K_SV_) and dissociation constant (K_d_) obtained by fluorescence quenching analysis of PDIA1 and PDIA3 in the presence of punicalagin. Data were calculated from fluorescence quenching analysis using both proteins (0.1 × 10^−6^ M) in reducing and non-reducing conditions (pH 7.4, 25 °C) and increasing concentration of punicalagin (up to 4 × 10^−6^ M). K_SV_ are reported as mean and SEM of at least three independent experiments.

	K_SV_ (M^−1^ × 10^3^)	K_d_ (M)
Reduced form		
PDIA1	97.9 ± 2.1	11.9 × 10^−6^
PDIA3	157.1 ± 1.9	10.0 × 10^−6^
Not reduced form		
PDIA1	183.2 ± 2.7	4.9 × 10^−6^
PDIA3	240.4 ± 3.6	3.9 × 10^−6^

**Table 2:** Thermodynamic data of the interaction between punicalagin and PDIAs. Both proteins (25 10^−6^ M) were analyzed in reduced form. Affinity constant (K_d_), molar enthalpy (ΔH), and entropy (TΔS) of the reaction at 25 °C were calculated processing data obtained from the isothermal titration.

	K_d_ (10^−6^ M)	ΔH (kcal/mol)	TΔS (kcal/mol)
PDIA1	1.0 ± 0.2	−2.7 ± 0.4	5.6 ± 0.5
PDIA3	1.2 ± 0.3	−1.1 ± 0.2	6.8 ± 0.4

## Data Availability

Data are contained within the article and the Supplementary Materials file.

## Supplementary Materials

The following are available online at https://www.mdpi.com/article/10.3390/biomedicines9111533/s1. Figure S1: Backbone RMSD over simulation time and KDE distribution plot of RMSD values. Figure S2: PDIA3 RMSD and RMSF domain-wise plots. Figure S3: PDIA1 RMSD and RMSF domain-wise plots. Figure S4: Distance between PDIA1_Ox_ and PDIA1_Red_ *a* and *a’* domains over time. Figure S5: PDIA1_Red_ in open and closed conformation. Figure S6: PCA representations of PDIA3 trajectories. Figure S7: PDIA3 conformations selected from MD simulations. Figure S8: PCA representations of PDIA1 trajectories. Figure S9: PDIA1 conformations selected from MD simulations. Figure S10: α and β-punicalagin in all-axial and all-equatorial conformations. Figure S11: Selected α and β-punicalagin binding modes for *a* and *a’* PDIA3 domains. Figure S12: Minimized binding modes of β-punicalagin on PDIA3 a domain. Figure S13: RMSD and RMSF plots comparing all PDIA3 trajectories. Figure S14: Selected α and β-punicalagin binding modes for *a* and *a’* PDIA1 domains. Figure S15: Selected α and β-punicalagin binding modes for *a’* PDIA1 domains displayed on the whole PDIA1 structure. Figure S16: Top-ranked binding mode of β-punicalagin on PDIA1 *a* domain. Figure S17: Lowest energy predicted binding mode for β-punicalagin on PDIA1 *a’* domain. Scheme S1: α and β-punicalagin 2D depictions. Figures S2, S3, S6, S7, S8, S9, S10, S12 and S16 are cited in the supplementary materials. References [3,16,37,40,46,47,48,49,50,51,52,53,54,55,56,57,58,59,60,61,62,63,64,65,66,67,68,69,70,71,72,73] are cited in the supplementary materials.

## Abbreviations

PDI	protein disulfide isomerase
IAV	influenza A virus
ITC	isothermal titration calorimetry
DSC	differential scanning calorimetry
MD	molecular dynamics
RMSD	root mean square deviation
KDE	kernel density estimation
MDCOM	mean distance between poses’ center of mass
SASA	solvent accessible surface area
RMSF	root mean square fluctuation
