# Supplementary material for: A Comparative Analysis of Punicalagin Interaction with PDIA1 and PDIA3 by Biochemical and Computational Approaches
Source: Biomedicines. 2021 Oct 25;9(11):1533. doi: 10.3390/biomedicines9111533 (PMC8614999; doi:10.3390/biomedicines9111533)
Supplement: Supplementary file 1 [file biomedicines-09-01533-s001.zip › biomedicines-1423987-supplementary.pdf]

Supplementary Materials

A Comparative Analysis of Punicalagin Interaction with PDIA1 and PDIA3 by Biochemical and Computational Approaches

Giuliano Paglia [1,†], Lorenzo Antonini [2,†], Laura Cervoni [1], Rino Ragno [2], Manuela Sabatino [2], Marco Minacori [1], Elisabetta Rubini [1,3] and Fabio Altieri [1,*]

[1] Department of Biochemical Sciences "A. Rossi Fanelli", Sapienza University of Rome, Piazzale Aldo Moro 5, 00185 Rome, Italy; giuliano.paglia@uniroma1.it (G.P.); laura.cervoni@uniroma1.it (L.C.); marco.minacori@uniroma1.it (M.M.); elisabetta.rubini@uniroma1.it (E.R.)
[2] Rome Center for Molecular Design, Department of Drug Chemistry and Technology, Sapienza University of Rome, Piazzale Aldo Moro 5, 00185 Rome, Italy; lorenzo.antonini@uniroma1.it (L.A.); rino.ragno@uniroma1.it (R.R.); manuela.sabatino@uniroma1.it (M.S.)
[3] Enrico ed Enrica Sovena Foundation, 00199 Rome, Italy
* Correspondence: fabio.altieri@uniroma1.it
† These authors contributed equally to this work.

S1. Computational Methods

S1.1. PDIAs Systems Construction

The three-dimensional structure of PDIA3 and PDIA1 were obtained from the Protein Data Bank (PDB ID: 3F8U, Tapasin/ERp57 heterodimer; PDB ID: 4EL1, Crystal Structure of Oxidized hPDI) [3,37]. Missing loops were filled using Modeller [46] and PDIA3 canonical protein sequence was restored using the mutation wizard implemented in Pymol [47] while residue protonation states were determined using PROPKA [48]. As the WCGHC active sites located in the a and a′ domains can be oxidized or reduced [49], both states were modeled: in the oxidized PDIA3 ($PDIA3_{Ox}$) the Cys57-Cys60 and Cys406-Cys409 cysteine couples were bound by a disulfide bridge, while in the reduced form ($PDIA3_{Red}$) the corresponding sulphur atoms were unbound and protonated. The same was done for PDIA1 oxidizing ($PDIA1_{Ox}$) and reducing ($PDIA1_{Red}$) the Cys53-Cys56 and Cys397-Cys400 cysteine couples. The PDIA3 starting structure found in 3F8U was complexed with tapasin, therefore the oxidized and reduced PDIA3 states were also modeled as complexes ($PDIA3_{Ox}$-Tap and $PDIA3_{Red}$-Tap). The four different systems were subjected to molecular dynamics (MD) simulations.

S1.2. Molecular Dynamics Simulations

All MD simulations were carried out by means of OpenMM [50] Python library, the Amber ff14SB [51] force field was used, the systems were solvated in a orthorhombic periodic box with OPC [52] four-point water model and neutralized adding Na+ or Cl- ions, the distance between the protein and the box boundary was set to 12 Å. The final topology and parameters files were prepared with tLeap from AmberTools suite (version 19) [53]. The electrostatic interactions were treated by Particle Mesh Ewald [54] algorithm and non-bonded interaction cutoff was set to 10 Å. Each MD simulation was preceded by a preparation protocol which included a solvent geometry minimization followed by a whole system minimization; then the minimized systems were subjected to 1 ns equilibration in an NVT ensemble during which the system was gradually heated to 300 K while applying a weak harmonic potential restrain to the solute molecule; a second equilibration of 1 ns is conducted in an NPT ensemble to relax the system to density of interest while applying a weak harmonic restraint to the solute. Finally, 100 ns production simulations were conducted at 1 atm and 300 K in an NPT ensemble. Pressure was controlled by a Monte Carlo barostat [55] while temperature was maintained constant using Langevin integration [56]. MD runs employed a 2 fs time step and trajectory snapshots were saved every 20 ps. Punicalagin-PDIA3 complex simulations were conducted with the same protocol; ligands' GAFF [57] parameters were calculated at the AM1-BCC [58] level of theory using antechamber [59].

S1.3. MD Analysis and Conformational Sampling

Trajectory analyses were accomplished using the MDTraj [60] and pytraj [53] Python libraries, while for statistical and numerical analysis the Scikit-Learn [61], SciPy [62] and Numpy [63] Python libraries were used. Particularly, the conformation sampling to use for molecular docking simulations from the trajectories was conducted using the k-means [64] clustering algorithm and the kernel density estimation (KDE) [65] method. While k-means clustering was used on the heavy atoms cartesian coordinates along the simulations, KDE was used on the first two principal component analysis (PCA) eigenvectors derived from cartesian coordinates to obtain the probability density function for each simulation frame.

S1.4. α. and β-Punicalagin Preparation

α and β-punicalagin three-dimensional structures were built using Open Babel [66]. All axial and all-equatorial conformers were modeled manually for α and β-punicalagin and geometrically optimized by energy minimization at the RM1 [67] semiempirical quantum mechanical level of theory using GAMESS [68]. The optimized geometries

were subjected to 100 ns MD simulation in explicit OPC water, using GAFF parameters calculated at the AM1-BCC level of theory using antechamber. The MD protocol was the same used for protein simulations, already described in Section S1.2. The resulting α and β-punicalagin trajectories were then analyzed and the conformations to use for docking simulations were sampled using the k-means clustering algorithm on heavy atoms cartesian coordinates along the simulation and KDE on the first two PCA eigenvectors derived from cartesian coordinates, similarly to the procedure used to sample protein conformations.

S1.5. Molecular Docking and Free Energy Calculations

Docking simulations were conducted using the Smina software by selecting the Vinardo scoring function [69] retaining 5 binding poses for each run. α and β-punicalagin conformations sampled from MD simulations were docked on protein conformations also taken from MD simulations. Ligands were docked on the a and a' PDIA3/PDIA1 domains and the two simulation boxes contained the whole a and a' domains, respectively. Among the predicted binding modes obtained from the docking for further analysis were selected 10 characterized by the best Vinardo score and 10 characterized by the highest structural correlation by means of probability density function for each binding mode. In particular, the latter selection was achieved using the first two eigenvectors retrieved by PCA calculated on predicted binding modes cartesian coordinates. The 20 selected binding modes were then merged with their locks, and the restating complexes were solvated and minimized similarly as above described. The minimized complexes were then used for ligand binding free energy calculations with the Molecular Mechanics/Generalized Born Surface Area (MM/GBSA) [40,70] method using the MMPBSA.py [71] python script available in the Ambertools suite.

S1.6. PDIA3-PDIA1 Comparison

PDIA3 and PDIA1 aminoacidic sequences were analyzed using the Biopython [72] Python library to obtain sequence alignments and sequence identities between full length sequence and between the *a* and *a'* domains. Alignment as well as sequence identities calculations were based on BLOSUM62 alignment matrix and results were also used with Pymol to visually display the conserved and non-conserved structural patterns.

S2. Computational Results

S2.1. PDIAs Molecular Dynamics Simulations Analysis

MD trajectories ($PDIA3_{Ox}$, $PDIA3_{Ox}$-Tap, $PDIA3_{Red}$, $PDIA3_{Red}$-Tap, $PDIA1_{Ox}$ and $PDIA1_{Red}$) were analyzed collecting the backbone RMSD along the simulations. PDIA3-Tapasin complexes ($PDIA3_{Ox}$-Tap and $PDIA3_{Red}$-Tap) showed lower RMSD values with respect to $PDIA3_{Ox}$ and $PDIA3_{Red}$. (Figure S1A) This difference is evidenced in the RMSD probability density function plot (Figure S1B). This was likely due to tapasin constraints on the movement of both PDIA3 *a* and *a'* domains. $PDIA1_{Ox}$ and $PDIA1_{Red}$ RMSD range and its trend is consistent with those observed in $PDIA3_{Ox}$ and $PDIA3_{Red}$ (Figure S1). However, $PDIA1_{Red}$ reached the equilibrium after 10 ns at an RMSD value of ~11 Å.

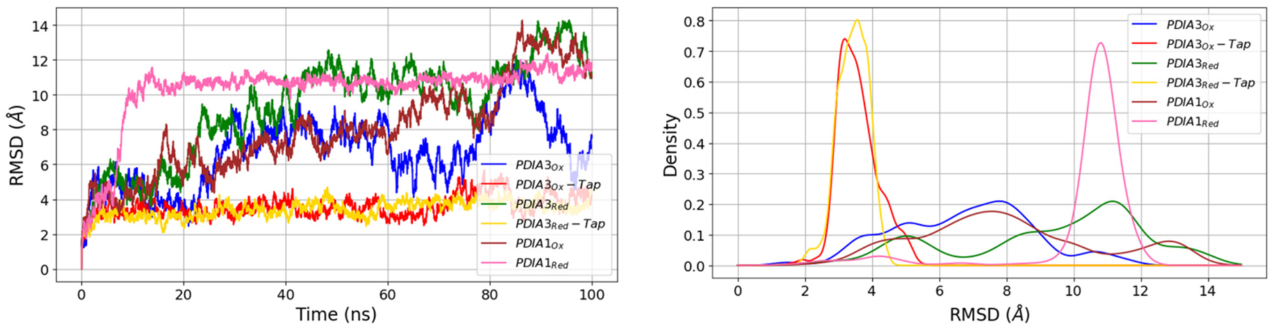

A **B**

Figure S1. (**A**) Backbone RMSD over simulation time plot; (**B**) KDE distribution plot of RMSD values.

To deeply evaluate PDIA3 stability and equilibration a domain wise RMSD and root mean square fluctuation (RMSF) calculations were used to investigate protein stability and flexibility and also to demonstrate the tapasin stabilization effect on the complex. Analysis of the corresponding plots (Figure S2) clearly indicated that the system stabilities were comparable as RMSD values were lower than 3 Å, suggesting that the four PDIA3 domains are steady structures. The RMSF analysis confirmed the previous observations returning low values, although with some exceptions. Higher RMSF indicated greater amplitude of backbone displacement for each residue during the simulation. The *a*, *b* and *b′* domains showed very similar RMSF trend except for some peaks corresponding to residues located in flexible loops. The *a′* domain RMSF plot showed higher flexibility for $PDIA3_{Ox}$ and $PDIA3_{Red}$ located nearby the WCGHC active sites, suggesting that tapasin might exert a stabilizing effect on this structural pattern. Indeed, the WCGHC pattern in both catalytic domains were in close contact with tapasin in the original crystal structure (3F8U). Moreover, other portions where $PDIA3_{Ox}$ and $PDIA3_{Red}$ exhibit higher RMSF values than $PDIA3_{Ox}$-Tap and $PDIA3_{Red}$-Tap were involved in the PDIA3-tapasin complex interactions.

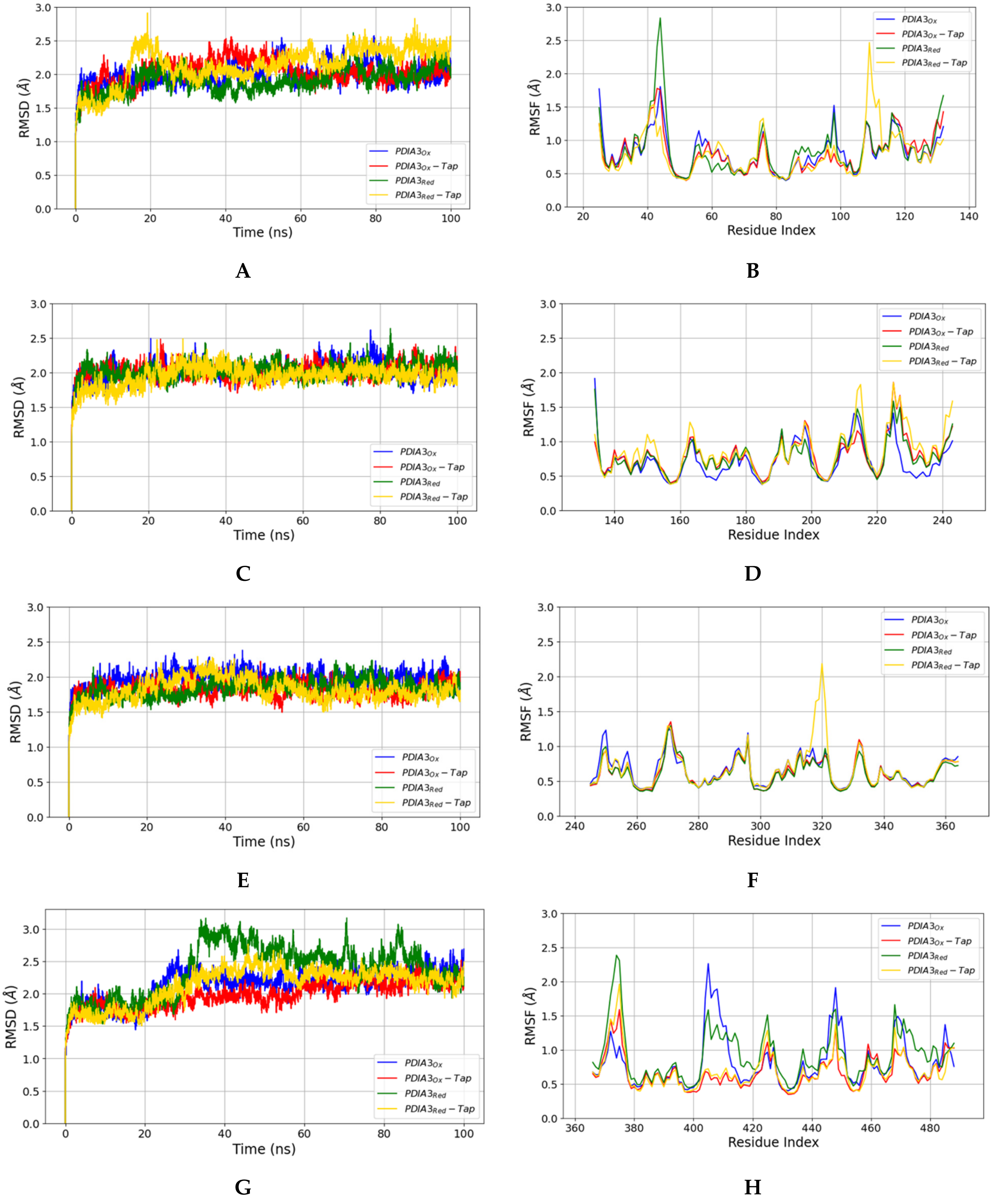

Figure S2. PDIA3 RMSD and RMSF domain-wise plots for: *a* domain (**A**,**B**), *b* domain (**C**,**D**), *b′* domain (**E**,**F**), *a′* domain (**G**,**H**).

Also, in the case of PDIA1 the *a* and *a′* domains' high mobility affects the analysis of protein stability and flexibility has to be investigated by means of domain-wise RMSD and RMSF plots. (Figure S3) As for PDIA3 these plots indicate that the PDIA1 domains are stable structures showing RMSD values always lower than 3 Å and the PDIA1$_{Ox}$ and PDIA1$_{Red}$ trends are always similar. RMSF showed comparable flexibility between PDIA1$_{Ox}$ and PDIA1$_{Red}$ domains.

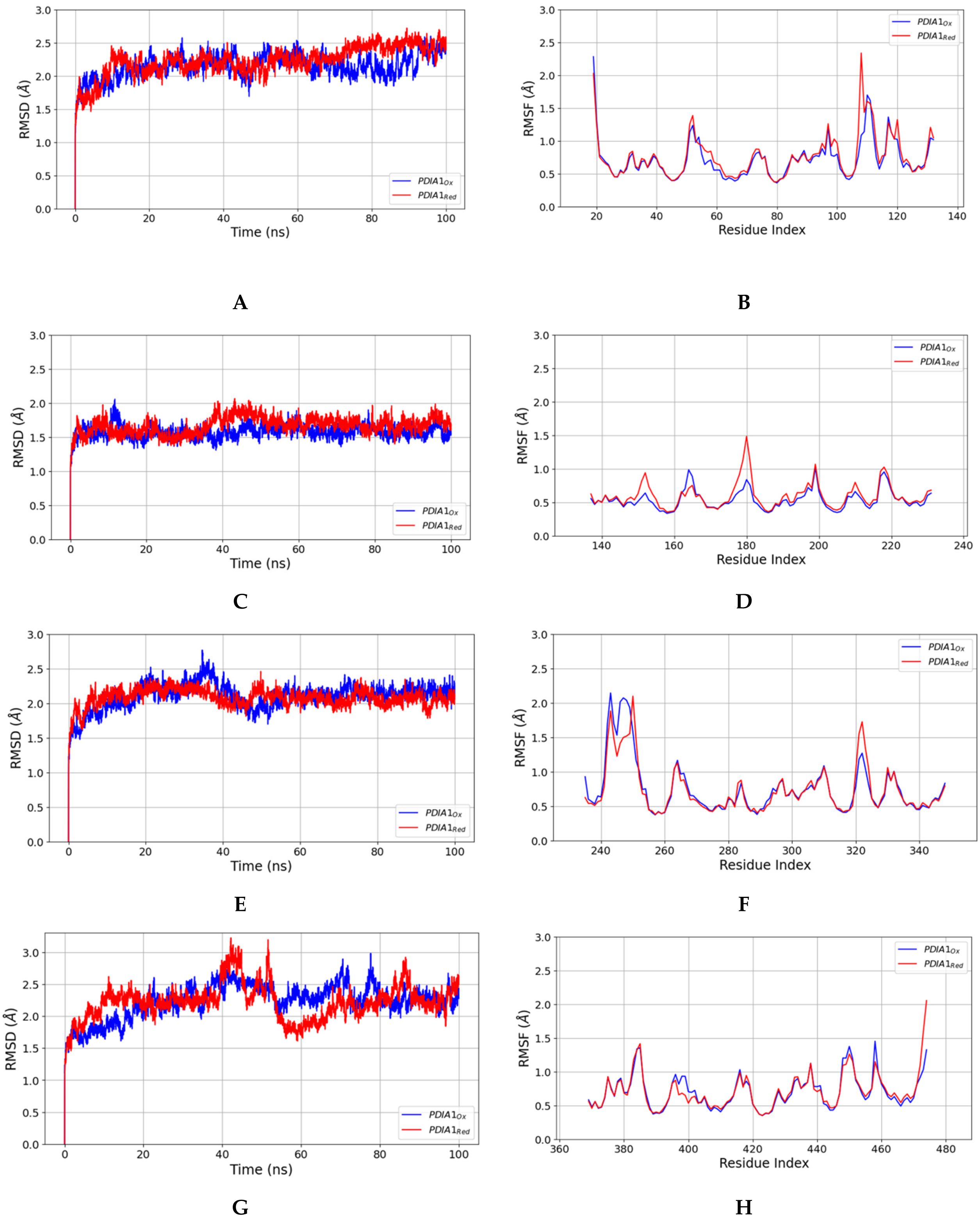

Figure S3. PDIA1 RMSD and RMSF domain-wise plots for: *a* domain (**A**,**B**), *b* domain (**C**,**D**), *b′* domain (**E**,**F**), *a′* domain (**G**,**H**).

To investigate on the PDIA1Red RMSD trend (Figure 1S) the distance between the *a* and *a′* domains along the simulations were collected (Figure S4). The values shows PDIA1Red switching from and open to a closed conformation during the first 10 ns (Figure S5), in agreement with experimental data that demonstrated the grater closeness between PDIA1 *a* and *a′* domains in the reduced form.[37]

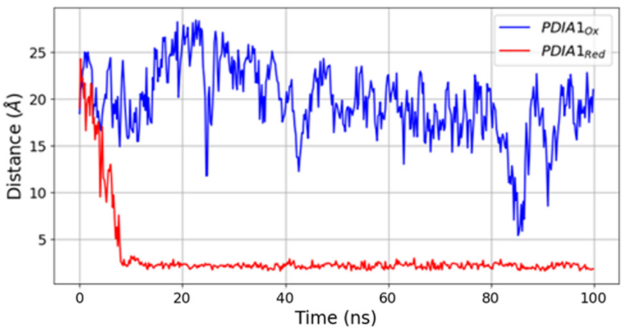

Figure S4. Distance between PDIA1Ox and PDIA1Red *a* and *a′* domains over time calculated along MD simulations.

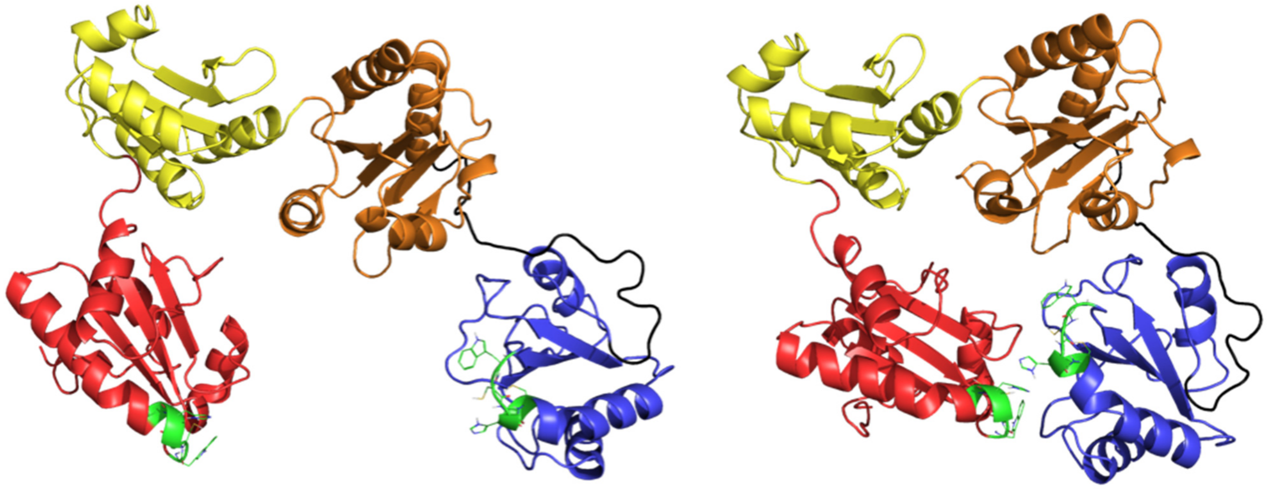

A **B**

Figure S5. PDIA1Red in "open" (**A**) and closed (**B**) conformation. PDIA1 *a* domain is depicted in red, *b* is yellow, *b′* is orange, *a′* is blue, *x* linker is black, WCGHC patterns are green lines.

S2.2. Conformation Sampling from PDIAs MD Simulations

In order to select PDIA3 conformation to be used in the docking simulations the MD trajectories were analyzed and a series of snapshots were sampled using different approaches as following: (1) *k*-means clustering on heavy atoms cartesian coordinates during the simulations; (2) *k*-means clustering on the above defined "active site" heavy atoms along the cartesian coordinates; (3) KDE on the first two PCA components derived from heavy atoms cartesian coordinates (Figure S6).

The k-means clustering algorithm was used to partition the trajectory snapshots in *k* groups, and once the clustering was completed the closest MD frames to the cluster centroids (red points in Figure S6A,C,D,F,G,I,J,L) were sampled from the trajectories and used for docking simulations. This method was also used on "active site", where "active site" was defined as the residues in a range of 10 Å from Trp56 and Trp405, in *a* and *a′* domains respectively, as from experimental evidences [16] punicalagin binding site should be located in proximity of these two residues. As the k-means clustering algorithm accomplished the partition in *k* clusters assigning each MD snapshot to the closest cluster centroid the closest MD snapshot to the centroid was likely the most representative structure of that cluster, and the sampled structures were likely to be very different from each other. The *k* value was chosen by means of silhouette analysis [73] on k-means clustering algorithm: the optimal identified *k* values ranged from 3 to 8. Based on this result a value of *k* equal to 5 was chosen for all simulations. For each trajectory 5 conformations were sampled using the above approaches 1 and 2 (*k* = 5). Differently KDE method was applied on the first two PCA components to obtain the probability density function for each frame, 5 snapshots with the highest value were sampled (red points in Figure S6B,E,H,K). Higher probability density function values indicate higher similarity and correlation between frames and the conformations sampled can be considered the most representative of each whole simulation. A total of 60 PDIA3 conformations (Figure S7) were thus sampled for the subsequent docking runs.

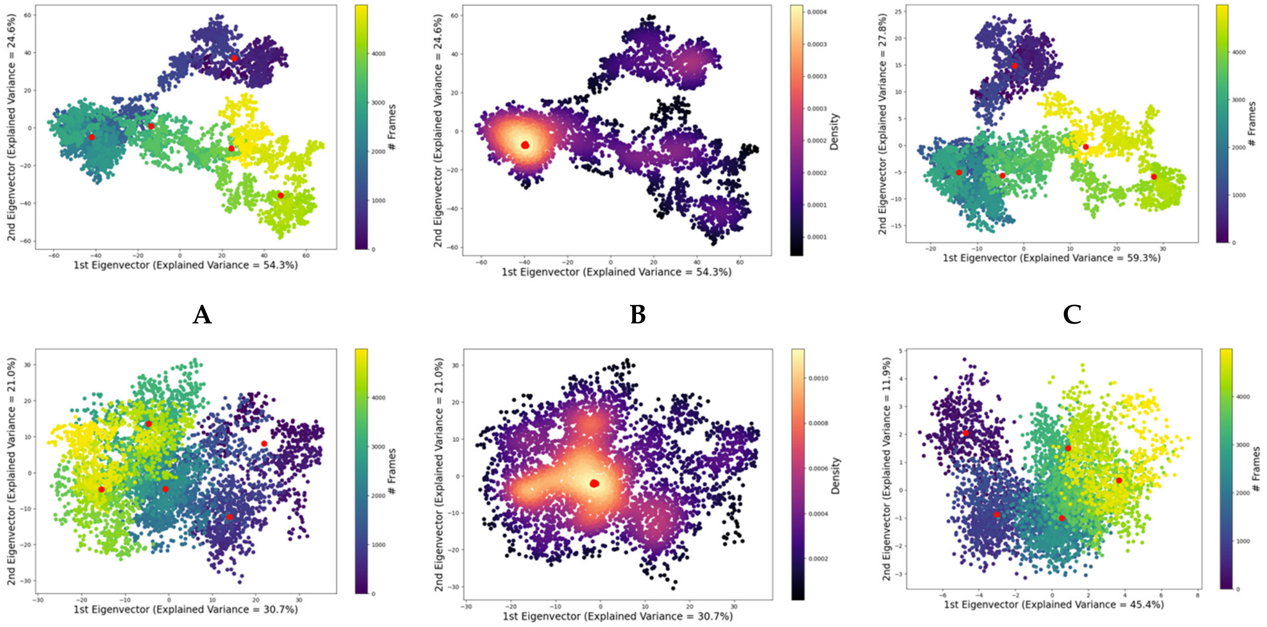

A **B** **C**

D **E** **F**

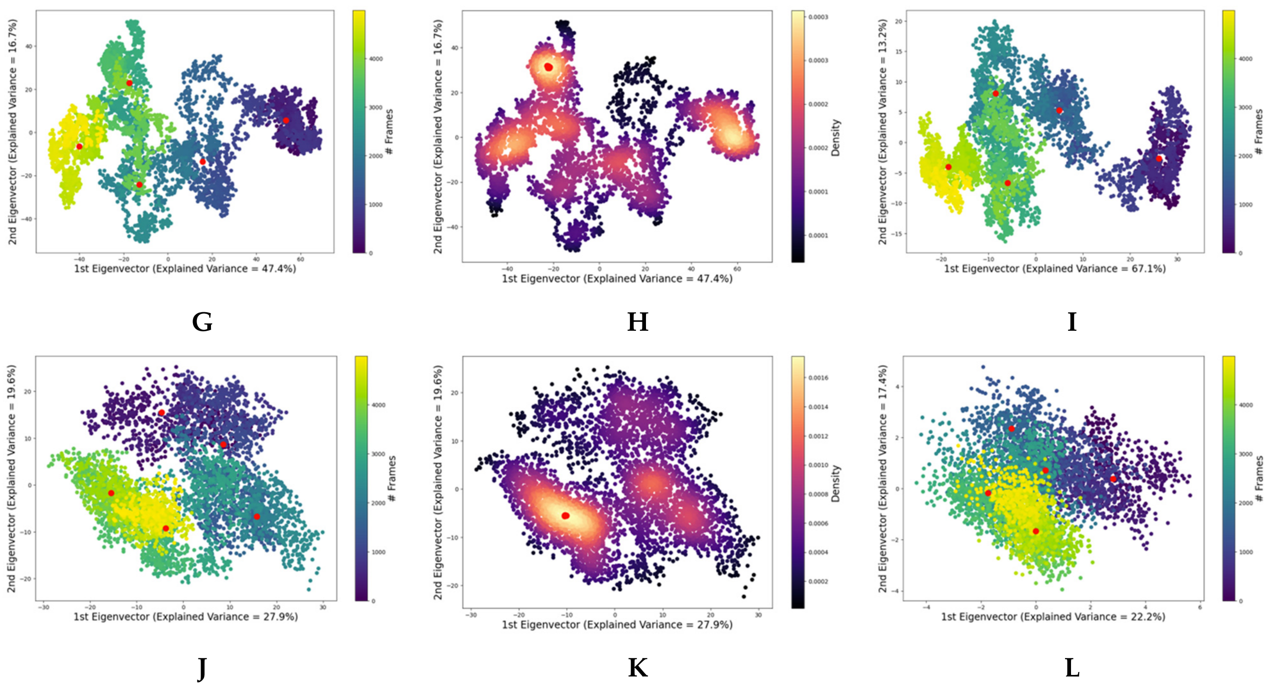

Figure S6. PCA representations of PDIA3 trajectories: dots are colored with respect to time (first and third columns), from blue to yellow, and density (second column), from black to yellow. The first row (**A–C**) is referred to PDIA3$_{Ox}$ trajectory, the second (**D–F**) to PDIA3$_{Ox}$-Tap, the third (**G–I**) to PDIA3$_{Red}$ and the fourth (**J–L**) to PDIA3$_{Red}$-Tap. In the first column (**A,D,G,J**) the PCA is calculated on the heavy atoms cartesian coordinates along the trajectories, the red dots are the k-means cluster centroids. In the second column (**B,E,H,K**) is reported the same PCA as for the first column but dots are colored with respect to probability density function, the red dost are the frames with the highest probability value. In the third column (**C,F,I,L**) is the depicted the PCA for "active site" cartesian coordinates along the simulations, the red dots are the k-means cluster centroids. On the plot axis are reported the explained variance for each eigenvector.

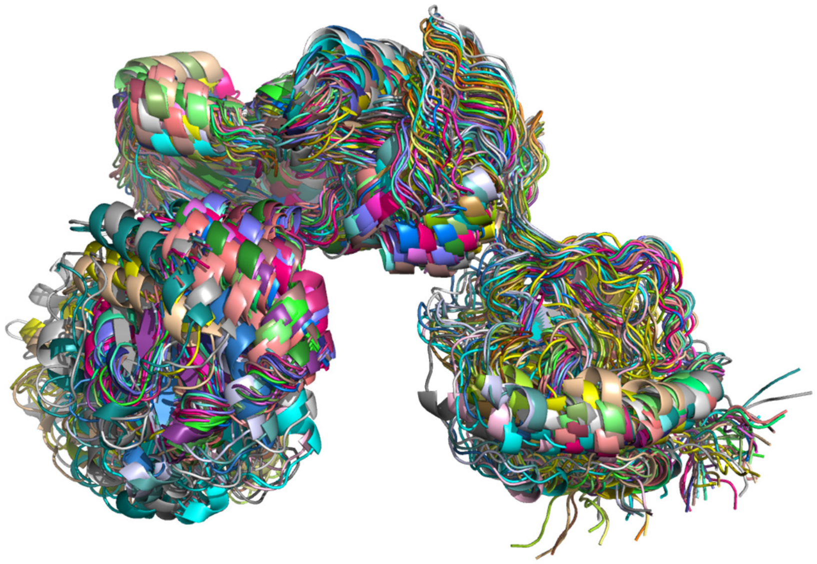

Figure S7. PDIA3 selected conformations from MD simulations.

To sample PDIA1 conformations for the subsequent docking simulations the PDIA1$_{Ox}$ and PDIA1$_{Red}$ trajectories were analyzed, and a series of snapshots were taken in the same fashion as above described for PDIA3. Trajectories were then analyzed with

the three approaches: k-means clustering on heavy atoms cartesian coordinates, k-means clustering on “active site” heavy atoms and KDE on the first two PCA components derived from heavy atoms cartesian coordinates. (Figure S8) Also, in this case the “active sites” was defined as the residues in a range of 10 Å from Trp52 and Trp396 in the *a* and *a′* domains, respectively. With each of the above-mentioned methods 5 simulation snapshots were sampled for a total of 30 PDIA1 conformations for subsequent docking runs. (Figure S9)

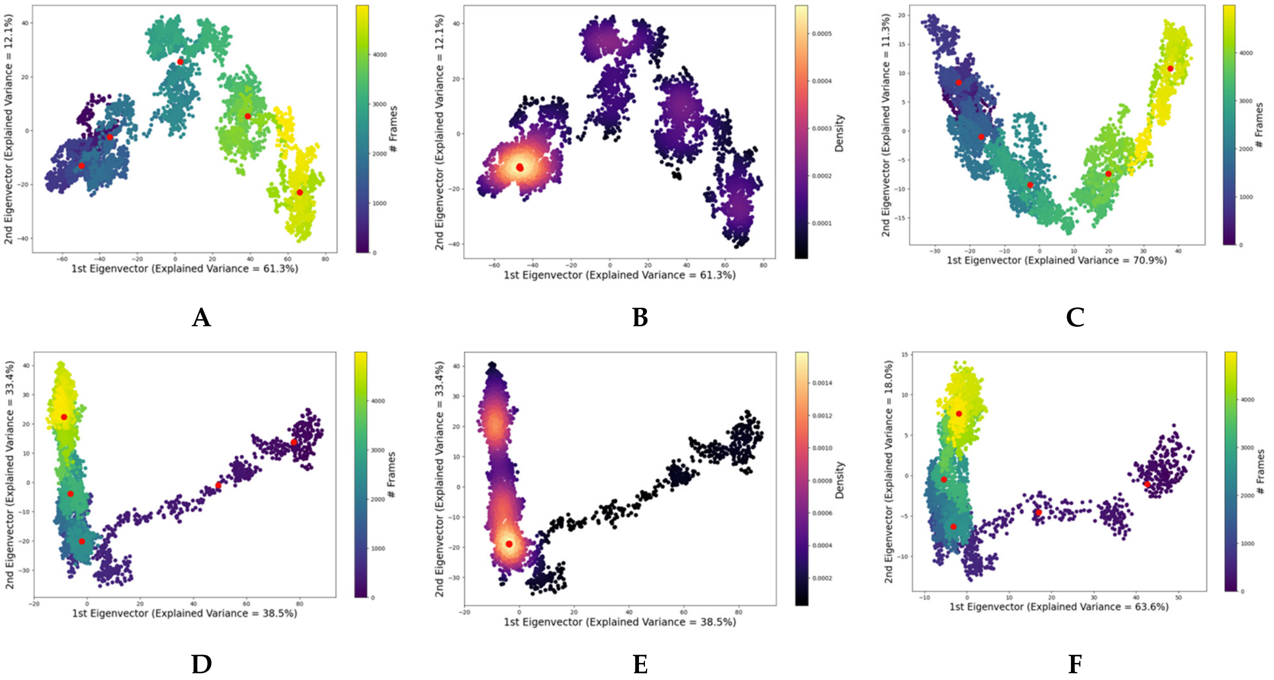

Figure S8. PCA representations of PDIA1 trajectories: dots are colored with respect to time (first and third columns), from blue to yellow, and density (second column), from black to yellow. The first row (**A–C**) is referred to PDIA1$_{Ox}$ trajectory, the second (**D–F**) to PDIA1$_{Red}$. In the first column (**A,D**) the PCA is calculated on the heavy atoms cartesian coordinates along the trajectories, the red dots are the k-means cluster centroids. In the second column (**B,E**) is reported the same PCA as for the first column but dots are colored with respect to probability density function, the red dost are the five with the highest probability value. In the third column (**C,F**) is the depicted the PCA for “active site” cartesian coordinates along the simulations, the red dots are the k-means cluster centroids. On the plot axis are reported the explained variance for each eigenvector.

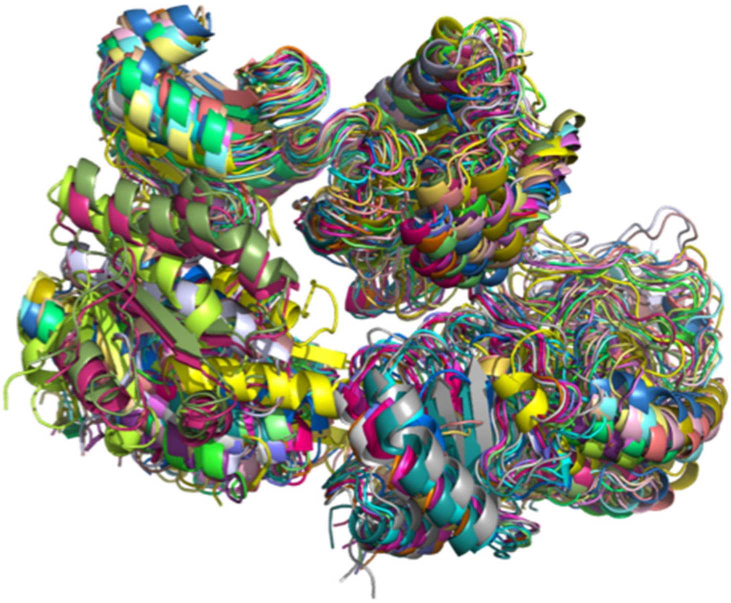

Figure S9. PDIA1 selected conformations from MD simulations.

S2.3. α. and β-Punicalagin MD Simulations

As α and β- are characterized by a cyclized highly constrained chemical structure and considering the smina limitations molecular docking were done using a rigid body docking procedure. To fulfill this lack of conformational flexibility, MD simulations of α and β-punicalagin were carried out to sample punicalagin conformations. The initial punicalagin structures were modeled in the all-axial and all-equatorial conformations and minimized at the RM1 level of theory. (Figure S10) As expected, all-equatorial conformations were the most stable for either the α or β epimers. In particular, β-punicalagin showed lower energies than α-punicalagin. The four structures were subjected to 100 ns MD simulation. Notably, for both α and β-punicalagin the all-axial conformations were converted to the all-equatorial ones within the first 5 ns of simulation in agreement with the semi-empirical quantum mechanical calculations. Punicalagin conformations were sampled using two of the three approaches above described for the PDIA3 conformation sampling: (1) *k*-means on heavy atoms cartesian coordinates; (2) KDE on first two PCA components derived from heavy atoms cartesian coordinates. Therefore, a total of 80 α and β-punicalagin conformations were selected for docking simulations.

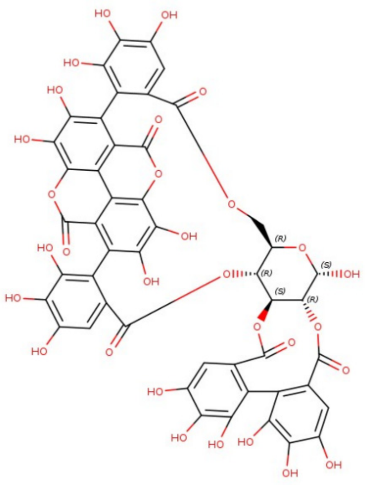

α-punicalagin

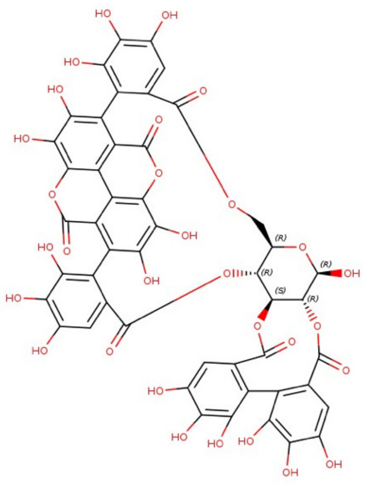

β-punicalagin

Scheme S1. α and β-punicalagin 2D depictions

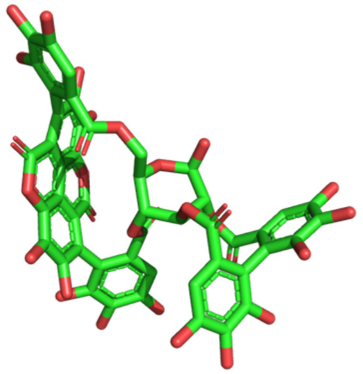

all-axial β-punicalagin

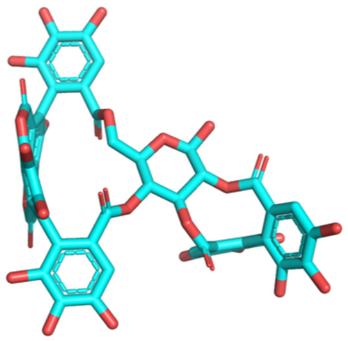

all-equatorial β-punicalagin

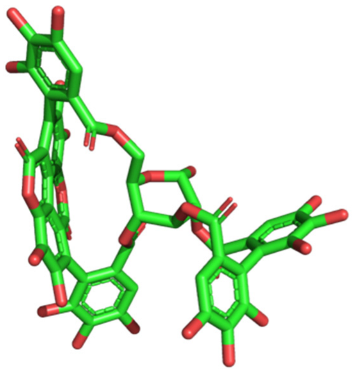

all-axial α-punicalagin

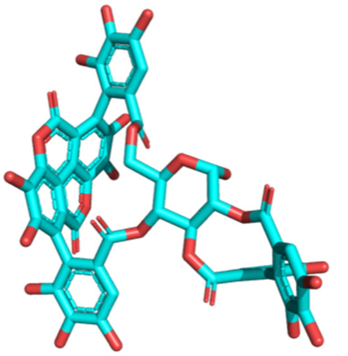

all-equatorial α-punicalagin

Figure S10. α and β-punicalagin in all-axial and all-equatorial conformations.

S2.4. PDIA3 Molecular Docking Simulations

The selected α and β-punicalagin conformations were docked onto the *a* and *a′* domains of the selected PDIA3 conformations returning 24,000 binding poses for each PDIA3 domain. Binding modes selection for further studies have been carried out basing on Vinardo score ranking and by statistical approach applying kernel density estimation (KDE) on poses′ heavy atoms cartesian coordinates first two principal components. The selected binding poses were used for free energy calculations for final rescoring and select the most likely binding conformation.

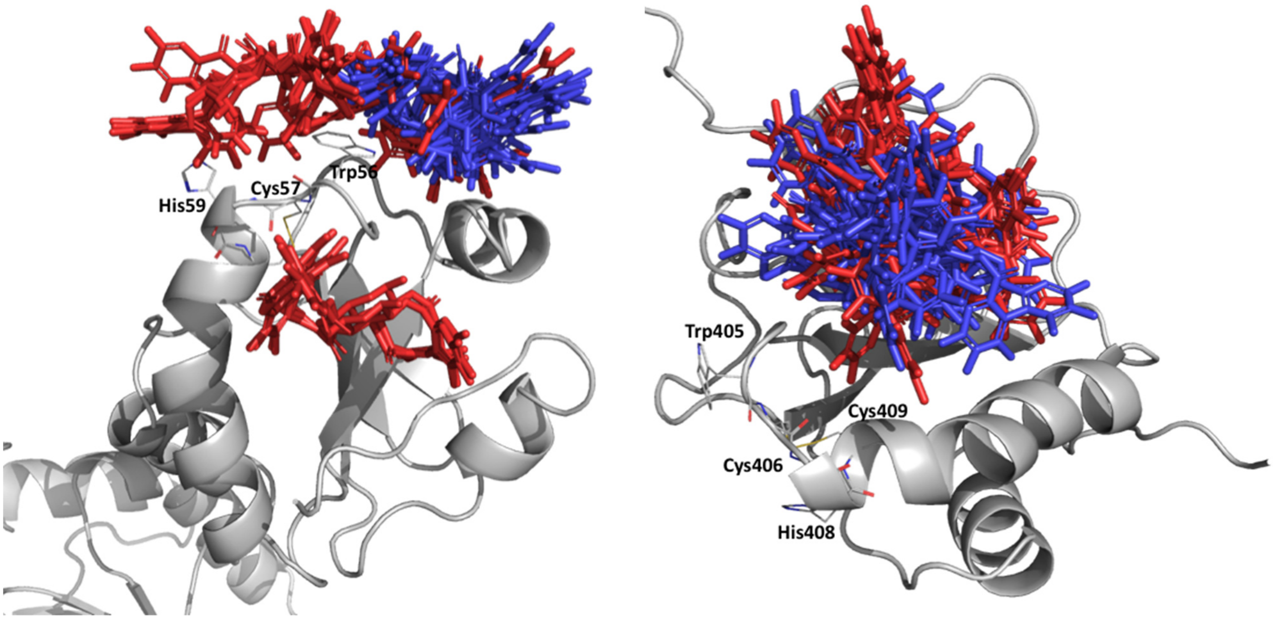

A **B**

Figure S11. Selected α and β-punicalagin binding modes for *a* (**A**) and *a′* (**B**) PDIA3 domains: conformation selected by Vinardo score are depicted in red, while those selected by structural correlation are depicted in blue.

S2.5. PDIA3 Free Energy Calculations

The docked keys were merged with the respective locks, then solvated, minimized subjected to free energy estimation by means of MM/GBSA calculations. Results evidenced the higher affinity of α and β-punicalagin for $PDIA3_{Ox}$, while *a′* seems to be the preferential binding domain. In both domains β-punicalagin showed higher affinity with respect to the α form. Results analysis started with ranking of binding modes by calculated free energy value: for the *a* domain the first two ranked binding modes (Figure S12A), −39.2 and −37.1 kcal mol^{-1} respectively, are almost identical while the third ranked (Figure S12B), −35.2 kcal mol^{-1}, is quite different since the ligand is settled on the other domain face; for the *a′* domain the top ranked binding mode (Figure 6A) is well detached from the other poses (−49.9 kcal mol^{-1}), showing a difference with the second ranked pose of ~8 kcal mol^{-1}.

α and β-punicalagin free energy results on the *a* domain displayed the first two ranked binding modes (Figure S12A), −39.2 and −37.1 kcal mol^{-1} respectively, being almost identical while the third ranked one (Figure S12B), −35.2 kcal mol^{-1}, is quite different since the ligand is settled on the other domain face; for the *a′* domain the top ranked binding mode (Figure 6A) is well detached from the other poses, showing a difference with the second ranked pose of ~8 kcal mol^{-1}.

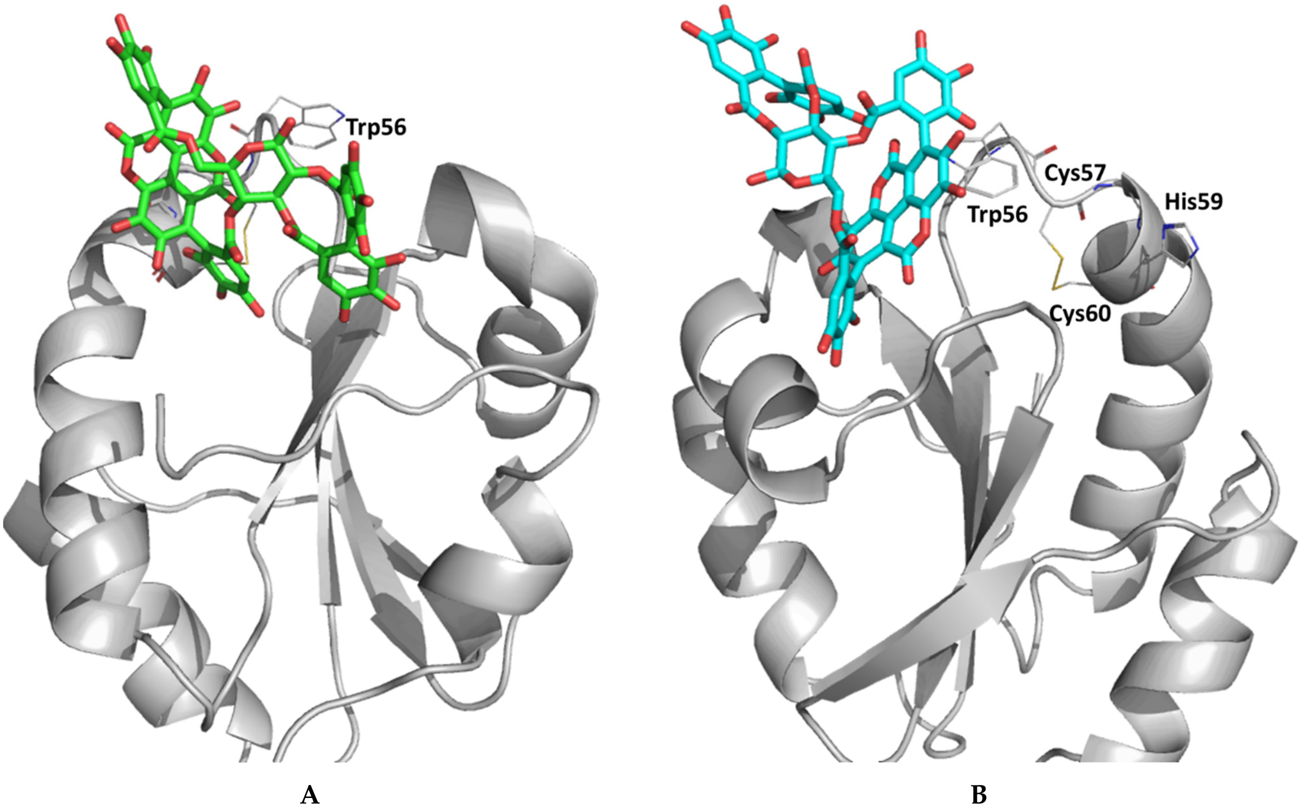

Figure S12. Minimized binding modes of β-punicalagin on PDIA3 *a* domain. (**A**) lowest energy predicted binding mode (−39.2 kcal mol^{-1}); (**B**) third ranked binding mode (−35.2 kcal mol^{-1}).

S2.6. MD Simulation on Best PDIA3-Punicalagin Complex

As the predicted binding mode was not found in close contact with quenching residue Trp405 and to understand how β-punicalagin could alter PDIA3 structure, a further analysis was conducted by means of 100 ns MD simulation. Trp405 solvent accessible surface area (SASA) collected along the simulation retunerd a different trend in comparison with PDIA3$_{Ox}$ and PDIA3$_{Red}$ simulations (Figure 7A). Indeed, the Trp405 SASA for the β-punicalagin complex (PDIA3$_{Ox}$-Pun) showed a Trp405 burial in the second half of the simulation due to the *a* and *a′* domains approaching each to the other to make contacts adopting a "closed" conformation (Figure 7C–D). RMSD analysis of PDIA3$_{Ox}$-Pun (Figure S13A) returned a trend consistent to PDIA3$_{Ox}$ and PDIA3$_{Red}$ while RMSF (Figure S13B) indicate a reduced flexibility of PDIA3$_{Ox}$-Pun complex with respect to PDIA3$_{Ox}$ and Red-PDIA3.

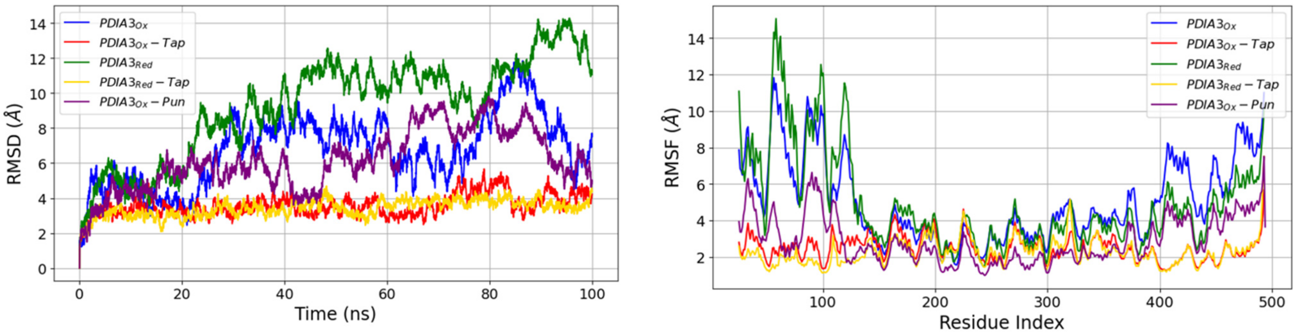

A **B**

Figure S13. RMSD (**A**) and RMSF (**B**) plots comparing all PDIA3 trajectories presented in this work including PDIA3$_{Ox}$-Pun.

S2.7. PDIA1 Molecular Docking Simulations

As for PDIA3, selected α and β-punicalagin conformations were docked into the *a* and *a′* domains of selected PDIA1 snapshots returning 12,000 binding poses for each PDIA1 catalytic domain. Binding modes selection for free energy calculations have been carried out basing on Vinardo score ranking and by statistical approach applying kernel density estimation (KDE) on poses' heavy atoms cartesian coordinates first two principal components, as already done for PDIA3. Selected docking results on the *a* domain (Figure S14A) showed two distinct conformations clusters matching the two selection approaches. Selected docking results on the *a′* domain (Figures S14B and S15) were mainly grouped into a cluster, except for few poses located at the *a′*/*b′* domains interface. The binding pocket in the *a′* domain mainly occupied by the most correlated binding poses (blue sticks in Figure S14B) was the same pocket identified as punicalagin binding site on PDIA3 *a′* domain, while the poses located at the *a′*/*b′* domains interface occupy a hydrophobic pocket only available in the $PDIA1_{Red}$ "closed" conformation. (Figure S15)

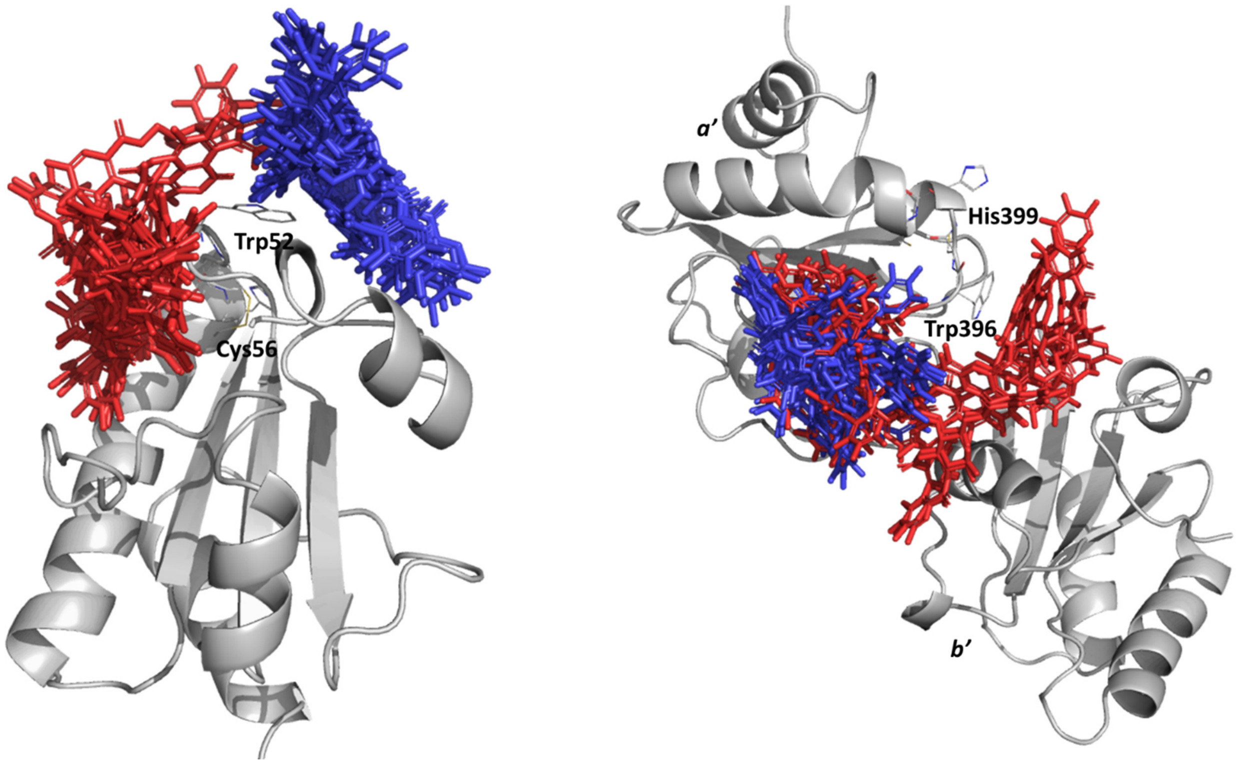

A **B**

Figure S14. Selected α and β-punicalagin binding modes for *a* (**A**) and *a′* (**B**) PDIA1 domains: conformation selected by Vinardo score are depicted in red, while those selected by structural correlation are depicted in blue.

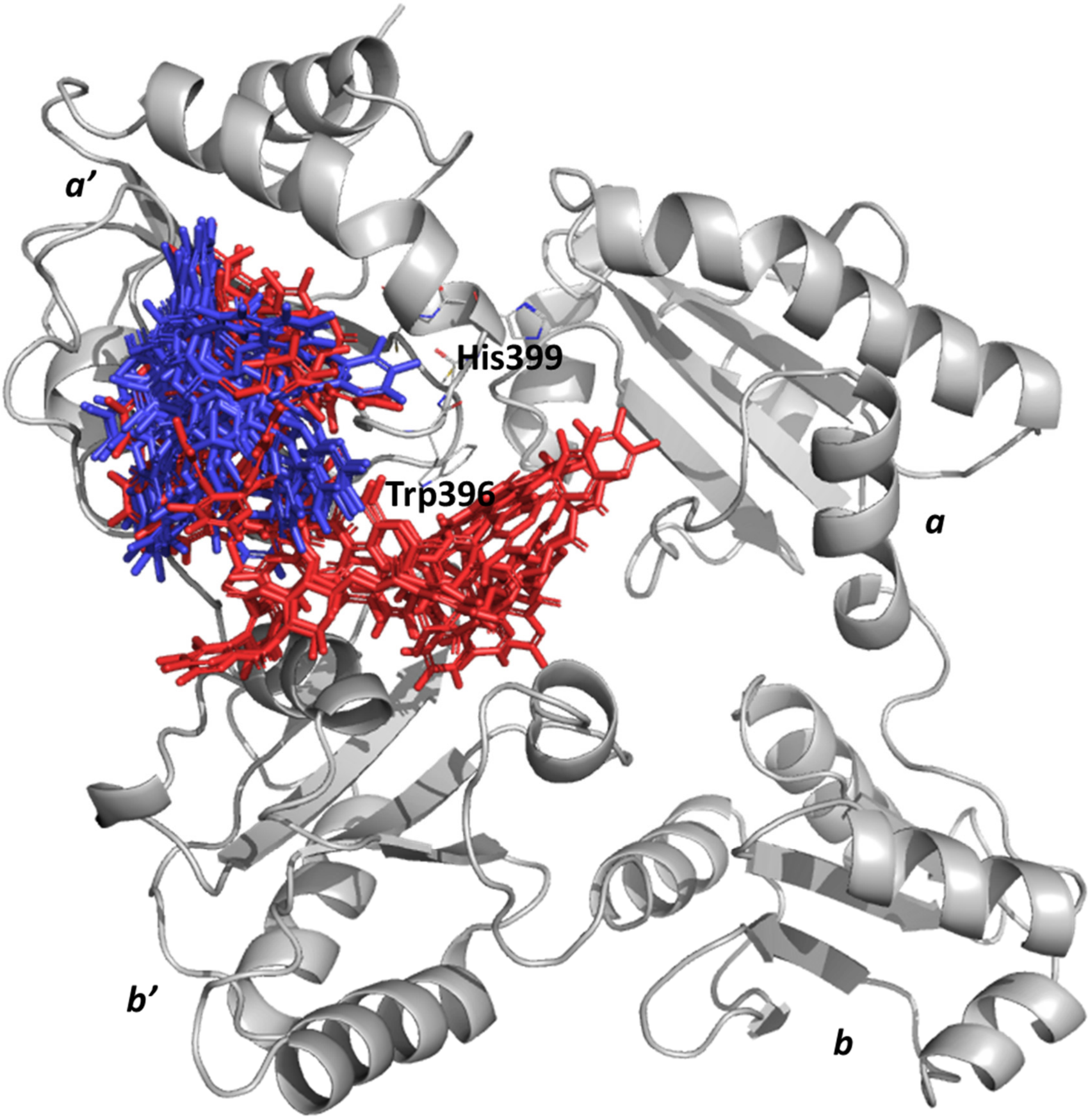

Figure S15. Selected α and β-punicalagin binding modes for *a′* PDIA1 domains displayed on the whole PDIA1 structure: conformation selected by Vinardo score are depicted in red, while those selected by structural correlation are depicted in blue.

S2.8. PDIA1 Free Energy Calculations

As for PDIA3, the selected PDIA1-punicalagin complexes for both the *a* and *a′* domains subjected to MM/PBSA free energy calculations. Results indicated *a′* as preferential punicalagin PDIA1 binding domain and β-punicalagin as favored epimer for both catalytic domains. Contrary to what seen for PDIA3, calculations evidenced higher punicalagin affinity for $PDIA1_{Red}$. The top ranked calculated binding free energy for β-punicalagin on PDIA1 *a* domain was −42.7 kcal mol^{-1} while its best binding free energy on the *a′* domain was −56.8 kcal mol^{-1}. The first five top ranked binding modes of β-punicalagin on PDIA1 *a* domain, lying in a range of ~5 kcal mol^{-1} were quite similar to each other and the first two (−42.7 and −42.2 kcal mol^{-1} respectively) were almost identical. The top ranked punicalagin binding mode (Figure S16) was associated with $PDIA1_{Ox}$ while the second with $PDIA1_{Red}$. Results for PDIA1 *a′* domain showed the top ranked binding mode (Figure S17) well detached from the other poses, with a difference of ~11 kcal mol^{-1} with the second one (−56.8 and −45.3 kcal mol^{-1} respectively). A β-punicalagin binding mode targeting the same pocket as for the best on $PDIA3_{Ox}$ was characterized by a binding free energy of −43.2 kcal mol^{-1}.

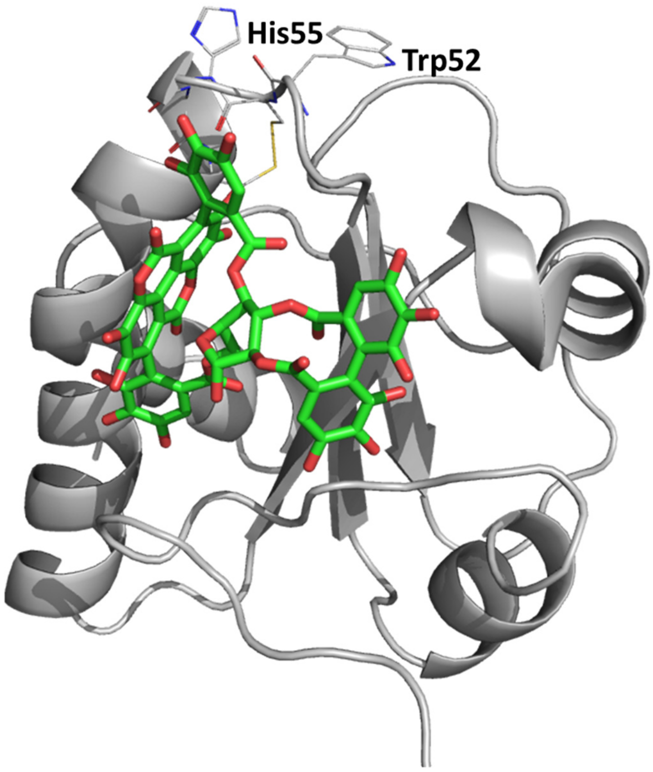

Figure S16. Top ranked binding mode of β-punicalagin on PDIA1 *a* domain (−42.7 kcal mol^{-1}).

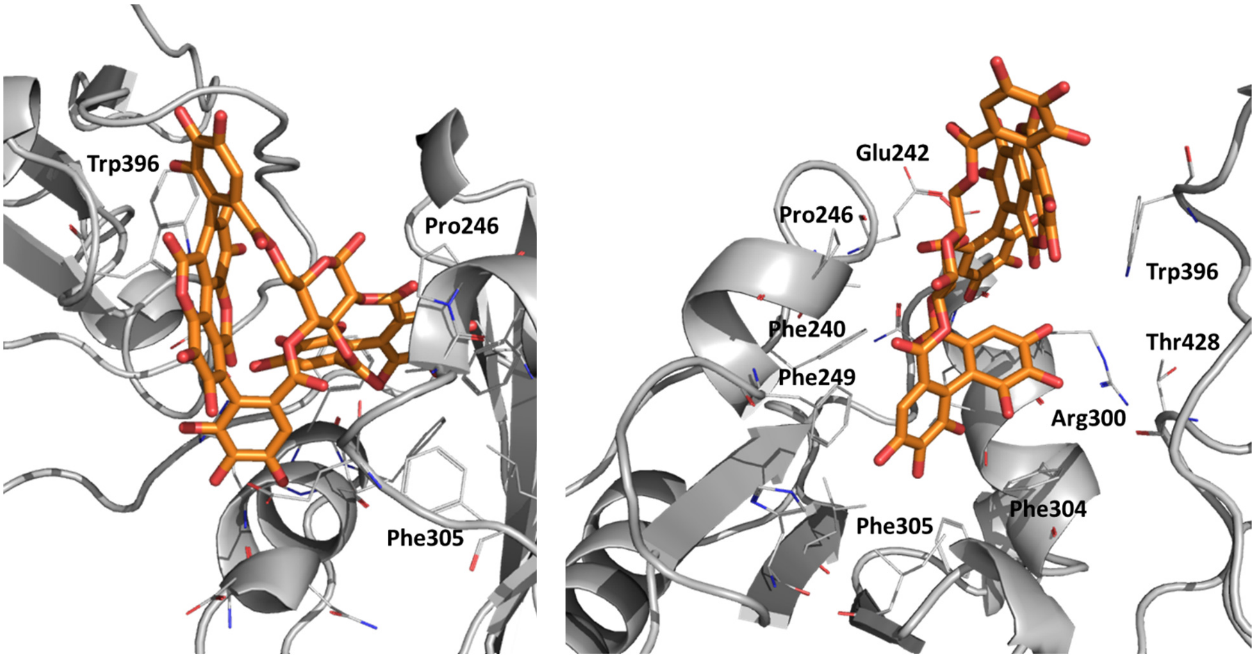

A **B**

Figure S17. Lowest energy predicted binding mode for β-punicalagin on PDIA1 *a′* domain. **A** and **B** show the same pose from two different views. Residues within a distance range of 5 Å are depicted as lines.

References

1. Hettinghouse, A.; Liu, R.; Liu, C. J. Multifunctional Molecule ERp57: From Cancer to Neurodegenerative Diseases. *Pharmacol Ther.* **2018**, 181, 34-48, doi:10.1016/j.pharmthera.2017.07.011
2. Frickel, E. M.; Frei, P.; Bouvier, M.; Stafford, W. F.; Helenius, A.; Glockshuber, R.; Ellgaard, L. ERp57 Is a Multifunctional Thiol-Disulfide Oxidoreductase. *J. Biol. Chem.* **2004**, 279 (18), 18277–18287, doi:10.1074/jbc.M314089200.
3. Dong, G.; Wearsch, P. A.; Peaper, D. R.; Cresswell, P.; Reinisch, K. M. Insights into MHC Class I Peptide Loading from the Structure of the Tapasin-ERp57 Thiol Oxidoreductase Heterodimer. *Immunity* **2009**, 30 (1), 21–32, doi:10.1016/j.immuni.2008.10.018.
4. Silvennoinen, L.; Myllyharju, J.; Ruoppolo, M.; Orrù, S.; Caterino, M.; Kivirikko, K. I.; Koivunen, P. Identification and Characterization of Structural Domains of Human ERp57: Association with Calreticulin Requires Several Domains. *J. Biol. Chem.* **2004**, 279(14), 13607-15, doi:10.1074/jbc.M313054200.
5. Turano, C.; Gaucci, E.; Grillo, C.; Chichiarelli, S. ERp57/GRP58: A Protein with Multiple Functions. *Cellular and Molecular Biology Letters* **2011**, 16, 539–563, doi:10.2478/s11658-011-0022-z.
6. Jessop, C. E.; Chakravarthi, S.; Garbi, N.; Hämmerling, G. J.; Lovell, S.; Bulleid, N. J. ERp57 Is Essential for Efficient Folding of Glycoproteins Sharing Common Structural Domains. *EMBO J.* **2007**, 26(1), 28-40, doi:10.1038/sj.emboj.7601505.
7. Santos, S. G.; Campbell, E. C.; Lynch, S.; Wong, V.; Antoniou, A. N.; Powis, S. J. Major Histocompatibility Complex Class I-ERp57-Tapasin Interactions within the Peptide-Loading Complex. *J. Biol. Chem.* **2007**, 282(24), 17587-17593, doi:10.1074/jbc.M702212200.
8. Nemere, I.; Garbi, N.; Hammerling, G.; Hintze, K. J. Role of the 1,25D 3-MARRS Receptor in the 1,25(OH) 2D 3-Stimulated Uptake of Calcium and Phosphate in Intestinal Cells. *Steroids* **2012**, 77(10), 897-902, doi:10.1016/j.steroids.2012.04.002.
9. Chichiarelli, S.; Gaucci, E.; Ferraro, A.; Grillo, C.; Altieri, F.; Cocchiola, R.; Arcangeli, V.; Turano, C.; Eufemi, M. Role of ERp57 in the Signaling and Transcriptional Activity of STAT3 in a Melanoma Cell Line. *Arch. Biochem. Biophys.* **2010**, 494(2), 178-183, doi:10.1016/j.abb.2009.12.004.
10. Chamberlain, N.; Korwin-Mihavics, B. R.; Nakada, E. M.; Bruno, S. R.; Heppner, D. E.; Chapman, D. G.; Hoffman, S. M.; van der Vliet, A.; Suratt, B. T.; Dienz, O.; Alcorn, J. F.; Anathy, V. Lung Epithelial Protein Disulfide Isomerase A3 (PDIA3) Plays an Important Role in Influenza Infection, Inflammation, and Airway Mechanics. *Redox Biol.* **2019**, 101129, doi:10.1016/j.redox.2019.101129.
11. Kondo, R.; Ishino, K.; Wada, R.; Takata, H.; Peng, W. X.; Kudo, M.; Kure, S.; Kaneya, Y.; Taniai, N.; Yoshida, H.; Naito, Z. Downregulation of Protein Disulfide-Isomerase A3 Expression Inhibits Cell Proliferation and Induces Apoptosis through STAT3 Signaling in Hepatocellular Carcinoma. *Int. J. Oncol*. **2019**, 54 (4), 1409–1421, doi:10.3892/ijo.2019.4710.
12. Li, S.; Zhao, X.; Chang, S.; Li, Y.; Guo, M.; Guan, Y. ERp57-Small Interfering RNA Silencing Can Enhance the Sensitivity of Drug-Resistant Human Ovarian Cancer Cells to Paclitaxel. *Int. J. Oncol.* **2019**, 54 (1), 249–260, doi:10.3892/ijo.2018.4628.
13. Ye, Q.; Fu, P.; Dou, J.; Wang, N. Downregulation of PDIA3 Inhibits Proliferation and Invasion of Human Acute Myeloid Leukemia Cells. *Onco. Targets. Ther*. **2018**, 11, 2925–2935, doi:10.2147/OTT.S162407.
14. Zou, H.; Wen, C.; Peng, Z.; Shao, Y. Y.; Hu, L.; Li, S.; Li, C.; Zhou, H. H. P4HB and PDIA3 Are Associated with Tumor Progression and Therapeutic Outcome of Diffuse Gliomas. *Oncol. Rep*. **2018**, 39 (2), 501–510, doi:10.3892/or.2017.6134.
15. Yang, Z.; Liu, J.; Shi, Q.; Chao, Y.; Di, Y.; Sun, J.; Zhang, J.; Huang, L.; Guo, H.; He, C. Expression of Protein Disulfide Isomerase A3 Precursor in Colorectal Cancer. *Onco. Targets. Ther*. **2018**, 11, 4159–4166, doi:10.2147/OTT.S154452.
16. Giamogante, F.; Marrocco, I.; Cervoni, L.; Eufemi, M.; Chichiarelli, S.; Altieri, F. Punicalagin, an Active Pomegranate Component, Is a New Inhibitor of PDIA3 Reductase Activity. *Biochimie* **2018**, 147, 122-129, doi:10.1016/j.biochi.2018.01.008.
17. Khatib, M.; Innocenti, M.; Giuliani, C.; Al- Tamimi, A.; Romani, A.; Mulinacci, N. Mesocarp and Exocarp of Laffan and Wonderful Pomegranate Varieties: By-Products as a Source of Ellagitannins. *Int. J. Food Nutr. Sci.* **2017**, 4 (1), 60–66, doi:10.15436/2377-0619.17.1465.
18. Chen, P. S.; Li, J. H.; Liu, T. Y.; Lin, T. C. Folk Medicine Terminalia Catappa and Its Major Tannin Component, Punicalagin, Are Effective against Bleomycin-Induced Genotoxicity in Chinese Hamster Ovary Cells. *Cancer Lett.* **2000**, 152 (2), 115–122, doi:10.1016/S0304-3835(99)00395-X.
19. Marzouk, M. S. A.; El-Toumy, S. A. A.; Moharram, F. A.; Shalaby, N. M. M.; Ahmed, A. A. E. Pharmacologically Active Ellagitannins from Terminalia Myriocarpa. *Planta Med.* **2002**, 68 (6), 523–527, doi:10.1055/s-2002-32549.
20. Asres, K.; Bucar, F.; Knauder, E.; Yardley, V.; Kendrick, H.; Croft, S. L. In Vitro Antiprotozoal Activity of Extract and Compounds from the Stem Bark of Combretum Molle. *Phyther. Res.* **2001**, 15 (7), 613–617, doi:10.1002/ptr.897.
21. Ismail, T.; Sestili, P.; Akhtar, S. Pomegranate Peel and Fruit Extracts: A Review of Potential Anti-Inflammatory and Anti-Infective Effects. *Journal of Ethnopharmacology* **2012**, 143(2), 397-405, doi:10.1016/j.jep.2012.07.004.
22. Turrini, E.; Ferruzzi, L.; Fimognari, C. Potential Effects of Pomegranate Polyphenols in Cancer Prevention and Therapy. *Oxidative Medicine and Cellular Longevity* **2015**, 2015:938475, doi:10.1155/2015/938475.
23. Wang, L.; Wang, X.; Wang, C. C. Protein Disulfide-Isomerase, a Folding Catalyst and a Redox-Regulated Chaperone. *Free Radic. Biol. Med.* **2015**, 83, 305–313, doi:10.1016/j.freeradbiomed.2015.02.007.
24. Kozlov, G.; Määttänen, P.; Thomas, D. Y.; Gehring, K. A Structural Overview of the PDI Family of Proteins. *FEBS Journal.* 277(19), 3924–3936, doi:10.1111/j.1742-4658.2010.07793.x.
25. Coppari, S.; Altieri, F.; Ferraro, A.; Chichiarelli, S.; Eufemi, M.; Turano, C. Nuclear Localization and DNA Interaction of Protein Disulfide Isomerase ERp57 in Mammalian Cells. *J. Cell. Biochem.* **2002**, 85(2), 325-333, doi:10.1002/jcb.10137.

26. Nguyen, V. D.; Saaranen, M. J.; Karala, A. R.; Lappi, A. K.; Wang, L.; Raykhel, I. B.; Alanen, H. I.; Salo, K. E. H.; Wang, C. C.; Ruddock, L. W. Two Endoplasmic Reticulum PDI Peroxidases Increase the Efficiency of the Use of Peroxide during Disulfide Bond Formation. *J. Mol. Biol.* **2011**, 406 (3), 503–515, doi:10.1016/j.jmb.2010.12.039.
27. Giamogante, F.; Marrocco, I.; Romaniello, D.; Eufemi, M.; Chichiarelli, S.; Altieri, F. Comparative Analysis of the Interaction between Different Flavonoids and PDIA3. *Oxid. Med. Cell. Longev*. **2016**, 2016:4518281, doi:10.1155/2016/4518281.
28. Raturi, A.; Mutus, B. Characterization of Redox State and Reductase Activity of Protein Disulfide Isomerase under Different Redox Environments Using a Sensitive Fluorescent Assay. *Free Radic. Biol. Med.* **2007**, 43(1), 62-70, doi:10.1016/j.freeradbiomed.2007.03.025.
29. Trnková, L.; Ricci, D.; Grillo, C.; Colotti, G.; Altieri, F. Green Tea Catechins Can Bind and Modify ERp57/PDIA3 Activity. Biochim. *Biophys. Acta - Gen. Subj.* **2013**, 1830(3), 2671-2682, doi:10.1016/j.bbagen.2012.11.011.
30. Ghisaidoobe, A. B. T.; Chung, S. J. Intrinsic Tryptophan Fluorescence in the Detection and Analysis of Proteins: A Focus on Förster Resonance Energy Transfer Techniques. *International Journal of Molecular Sciences.* **2014**, 15(12), 22518-22538, doi:10.3390/ijms151222518.
31. Lakowicz, J. R.; Weber, G. Quenching of Fluorescence by Oxygen. Probe for Structural Fluctuations in Macromolecules. *Biochemistry* **1973**, 12(21), 4161-4170, doi:10.1021/bi00745a020.
32. Lakowicz, J.R. Principles of Fluorescence Spectroscopy; Springer: New York, NY, USA, 2006; Quenching of Fluorescence, pp. 277-330, doi:10.1007/978-0-387-46312-4.
33. Ware, W. R. Oxygen Quenching of Fluorescence in Solution: An Experimental Study of the Diffusion Process. *J. Phys. Chem.* **1962,** 66 (3), 455-458, doi:10.1021/j100809a020.
34. Bi, S.; Song, D.; Tian, Y.; Zhou, X.; Liu, Z.; Zhang, H. Molecular Spectroscopic Study on the Interaction of Tetracyclines with Serum Albumins. *Spectrochim. Acta - Part A Mol. Biomol. Spectrosc.* **2005**, 61(4), 629-636, doi:10.1016/j.saa.2004.05.028.
35. Sun, Y.; Zhang, H.; Sun, Y.; Zhang, Y.; Liu, H.; Cheng, J.; Bi, S.; Zhang, H. Study of Interaction between Protein and Main Active Components in Citrus Aurantium L. by Optical Spectroscopy. *J. Lumin.* **2010**, 130 (2), 270-279, doi:10.1016/j.jlumin.2009.09.002.
36. Du, X.; Li, Y.; Xia, Y. L.; Ai, S. M.; Liang, J.; Sang, P.; Ji, X. L.; Liu, S. Q. Insights into Protein–Ligand Interactions: Mechanisms, Models, and Methods. *International Journal of Molecular Sciences* **2016**, 17(2), 144, doi:10.3390/ijms17020144.
37. Wang, C.; Li, W.; Ren, J.; Fang, J.; Ke, H.; Gong, W.; Feng, W.; Wang, C. C. Structural Insights into the Redox-Regulated Dynamic Conformations of Human Protein Disulfide Isomerase. *Antioxidants and Redox Signaling*. **2013**, 19(1), 36-45, doi:10.1089/ars.2012.4630.
38. Khan, H. A.; Mutus, B. Protein Disulfide Isomerase a Multifunctional Protein with Multiple Physiological Roles. *Frontiers in Chemistry* **2014**, 2, 70, doi:10.3389/fchem.2014.00070.
39. Ragno, R.; Frasca, S.; Manetti, F.; Brizzi, A.; Massa, S. HIV-Reverse Transcriptase Inhibition: Inclusion of Ligand-Induced Fit by Cross-Docking Studies. *J. Med. Chem*. **2005**, 48 (1), 200–212, doi:10.1021/jm0493921.
40. Kollman, P. A.; Massova, I.; Reyes, C.; Kuhn, B.; Huo, S.; Chong, L.; Lee, M.; Lee, T.; Duan, Y.; Wang, W.; Donini, O.; Cieplak, P.; Srinivasan, J.; Case, D. A.; Cheatham, T. E. Calculating Structures and Free Energies of Complex Molecules: Combining Molecular Mechanics and Continuum Models. *Acc. Chem. Res.* **2000**, 33 (212), 889-897. https://doi.org/10.1021/ar000033j.
41. Bastos, T. M.; Botelho, M.; Soares, P.; Franco, C. H.; Alc, L.; Antonini, L.; Sabatino, M.; Mautone, N.; Freitas-junior, L. H.; Moraes, C. B.; Ragno, R.; Rotili, D.; Schenkman, S.; Mai, A.; Moretti, N. S. Identification of Inhibitors to Trypanosoma Cruzi Sirtuins Based on Compounds Developed to Human Enzymes. *Int J Mol Sci.* **2020**, 21(10), 3659, doi:10.3390/ijms21103659.
42. Freedman, R. B.; Desmond, J. L.; Byrne, L. J.; Heal, J. W.; Howard, M. J.; Sanghera, N.; Walker, K. L.; Wallis, A. K.; Wells, S. A.; Williamson, R. A.; Römer, R. A. 'Something in the Way She Moves': The Functional Significance of Flexibility in the Multiple Roles of Protein Disulfide Isomerase (PDI). *Biochim. Biophys. Acta - Proteins Proteomics* **2017**, 1865 (11), 1383–1394, doi:10.1016/j.bbapap.2017.08.014.
43. Römer, R. A.; Wells, S. A.; Emilio Jimenez-Roldan, J.; Bhattacharyya, M.; Vishweshwara, S.; Freedman, R. B. The Flexibility and Dynamics of Protein Disulfide Isomerase. *Proteins Struct. Funct. Bioinforma*. **2016**, 84 (12), 1776–1785, doi:10.1002/prot.25159.
44. Wang, C.; Chen, S.; Wang, X.; Wang, L.; Wallis, A. K.; Freedman, R. B.; Wang, C. C. Plasticity of Human Protein Disulfide Isomerase: Evidence for Mobility around the x-Linker Region and Its Functional Significance. *J. Biol. Chem*. **2010**, 285 (35), 26788–26797, doi:10.1074/jbc.M110.107839.
45. Guyette, J.; Evangelista, B.; Tatulian, S. A.; Teter, K. Stability and Conformational Resilience of Protein Disulfide Isomerase. *Biochemistry* **2019**, 58 (34), 3572–3584, doi:10.1021/acs.biochem.9b00405.
46. Webb, B.; Sali, A. Comparative protein structure modeling using MODELLER. *Curr. Protoc. Bioinforma.* **2016**, *54*, 5.6.1-5.6.37.
47. *The PyMOL Molecular Graphics System. Version 2.0*; Schrödinger, LLC: New York, NY, USA.
48. Olsson, M. H. M.; SØndergaard, C. R.; Rostkowski, M.; Jensen, J. H. PROPKA3: Consistent Treatment of Internal and Surface Residues in Empirical p K a Predictions. *J. Chem. Theory Comput.* **2011**, 7 (2), 525–537. https://doi.org/10.1021/ct100578z.
49. Darby, N. J.; Creighton, T. E. Functional Properties of the Individual Thioredoxin-like Domains of Protein Disulfide Isomerase. *Biochemistry* **1995**, 34 (37), 11725-35. https://doi.org/10.1021/bi00037a009.
50. Eastman, P.; Swails, J.; Chodera, J. D.; McGibbon, R. T.; Zhao, Y.; Beauchamp, K. A.; Wang, L. P.; Simmonett, A. C.; Harrigan, M. P.; Stern, C. D.; Wiewiora, R. P.; Brooks, B. R.; Pande, V. S. OpenMM 7: Rapid Development of High Performance Algorithms for Molecular Dynamics. *PLoS Comput. Biol.* **2017**, 13 (7), 1–17. https://doi.org/10.1371/journal.pcbi.1005659.

51. Maier, J. A.; Martinez, C.; Kasavajhala, K.; Wickstrom, L.; Hauser, K. E.; Simmerling, C. Ff14SB: Improving the Accuracy of Protein Side Chain and Backbone Parameters from Ff99SB. *J. Chem. Theory Comput.* **2015**, 11, 8, 3696–3713. https://doi.org/10.1021/acs.jctc.5b00255.
52. Izadi, S.; Anandakrishnan, R.; Onufriev, A. V. Building Water Models: A Different Approach. *J. Phys. Chem. Lett.* **2014**, 5, 21, 3863–3871. https://doi.org/10.1021/jz501780a.
53. D.A. Case, I.Y. Ben-Shalom, S.R. Brozell, D.S. Cerutti, T.E. Cheatham, III, V.W.D. Cruzeiro, T.A. Darden, R.E. Duke, D. Ghoreishi, G. Giambasu, et al. **2019**, *Amber* 2019, University of California, San Francisco, CA, USA, 2019.
54. Darden, T.; York, D.; Pedersen, L. Particle Mesh Ewald: An N·log(N) Method for Ewald Sums in Large Systems. *J. Chem. Phys.* **1993**, 18, 10089-10092. https://doi.org/10.1063/1.464397.
55. Chow, K. H.; Ferguson, D. M. Isothermal-Isobaric Molecular Dynamics Simulations with Monte Carlo Volume Sampling. *Comput. Phys. Commun.* **1995**, 91 (1-3), 283-289. https://doi.org/10.1016/0010-4655(95)00059-O.
56. Lzaguirre, J. A.; Catarello, D. P.; Wozniak, J. M.; Skeel, R. D. Langevin Stabilization of Molecular Dynamics. *J. Chem. Phys.* **2001**, 144, 2090. https://doi.org/10.1063/1.1332996.
57. Wang, J.; Wolf, R. M.; Caldwell, J. W.; Kollman, P. A.; Case, D. A. Development and testing of a general Amber force field. *J. Comput. Chem* **2004**, 25 (9), 1157-1174. doi:10.1002/jcc.20035
58. Jakalian, A.; Bush, B. L.; Jack, D. B.; Bayly, C. I. Fast, Efficient Generation of High-Quality Atomic Charges. AM1-BCC Model: I. Method. *J. Comput. Chem.* **2000**, 21 (2) : 132-146. https://doi.org/10.1002/(SICI)1096-987X(20000130)21:2<132::AID-JCC5>3.0.CO;2-P.
59. Wang, J.; Wang, W.; Kollman, P. A.; Case, D. A. Automatic atom type and bond type perception in molecular mechanical calculations. *J. Mol. Graph. Model.*, **2006**, 25 (2), 247-260. https://10.1016/j.jmgm.2005.12.005.
60. McGibbon, R. T.; Beauchamp, K. A.; Harrigan, M. P.; Klein, C.; Swails, J. M.; Hernández, C. X.; Schwantes, C. R.; Wang, L. P.; Lane, T. J.; Pande, V. S. MDTraj: A Modern Open Library for the Analysis of Molecular Dynamics Trajectories. *Biophys. J.* **2015**, 109 (8), 1528-1532. https://doi.org/10.1016/j.bpj.2015.08.015.
61. Pedregosa, F.; Varoquaux, G.; Gramfort, A.; Michel, V.; Thirion, B.; Grisel, O.; Blondel, M.; Prettenhofer, P.; Weiss, R.; Dubourg, V.; Vanderplas, J.; Passos, A.; Cournapeau, D.; Brucher, M.; Perrot, M.; Duchesnay, É. Scikit-Learn: Machine Learning in Python. *J. Mach. Learn. Res.* **2011**, 12, 2825-2830.
62. Virtanen, P.; Gommers, R.; Oliphant, T.E., Haberland, M.; Reddy, T.; Cournapeau, D.; Burovski, E.; Peterson, P.; Weckesser, W.; Bright, J.; et al. SciPy 1.0: fundamental algorithms for scientific computing in Python. *Nat Methods.* **2020**, 17, 261–272. https://doi.org/10.1038/s41592-019-0686-2.
63. Harris, C.R.; Millman, K. J.; vad der Walt, S. J., Gommers, R.; Virtanen, P.; Cournapeau, D.; Wieser, E.; Taylor, J.; Berg, S.; Smith, N.J.; et al. Array programming with NumPy. *Nature*, **2020**. 585, 357–362. https://doi.org/10.1038/s41586-020-2649-2.
64. Lloyd, S. P. Least Squares Quantization in PCM. *IEEE Trans. Inf. Theory* **1982**, 18 (2), 129-137. https://doi.org/10.1109/TIT.1982.1056489.
65. Scott, D. W. *Multivariate Density Estimation: Theory, Practice, and Visualization: Second Edition*; John Wiley & Sons, Inc.: Hoboken, NJ, USA, 2015. https://doi.org/10.1002/9781118575574.
66. O'Boyle, N. M.; Banck, M.; James, C. A.; Morley, C.; Vandermeersch, T.; Hutchison, G. R. Open Babel: An Open Chemical Toolbox. *J. Cheminform.* **2011**, 3, 33. https://doi.org/10.1186/1758-2946-3-33.
67. Rocha, G. B.; Freire, R. O.; Simas, A. M.; Stewart, J. J. P. RM1: A Reparameterization of AM1 for H, C, N, O, P, S, F, Cl, Br, and I. *J. Comput. Chem.* **2006**, 27 (10),1101-1111. https://doi.org/10.1002/jcc.20425.
68. Schmidt, M. W.; Baldridge, K. K.; Boatz, J. A.; Elbert, S. T.; Gordon, M. S.; Jensen, J. H.; Koseki, S.; Matsunaga, N.; Nguyen, K. A.; Su, S.; Windus, T. L.; Dupuis, M.; Montgomery, J. A. General Atomic and Molecular Electronic Structure System. *J. Comput. Chem.* **1993**, 14 (11), 1347-1363. https://doi.org/10.1002/jcc.540141112.
69. Quiroga, R.; Villarreal, M. A. Vinardo: A Scoring Function Based on Autodock Vina Improves Scoring, Docking, and Virtual Screening. *PLoS One* **2016,** *11*, e0155183. https://doi.org/10.1371/journal.pone.0155183.
70. Hou, T.; Wang, J.; Li, Y.; Wang, W. Assessing the Performance of the MM/PBSA and MM/GBSA Methods. 1. The Accuracy of Binding Free Energy Calculations Based on Molecular Dynamics Simulations. *J. Chem. Inf. Model.* **2011**, 51 (1), 69-82. https://doi.org/10.1021/ci100275a.
71. Miller, B. R., 3rd; McGee, T. D., Jr.; Swails, J. M.; Homeyer, N.; Gohlke, H.; Roitberg, A. E., MMPBSA.py: An Efficient Program for End-State Free Energy Calculations. *J. Chem. Theory Comput.* **2012**, 8 (9), 3314-21. https://doi.org/10.1021/ct300418h.
72. Cock, P. J. A.; Antao, T.; Chang, J. T.; Chapman, B. A.; Cox, C. J.; Dalke, A.; Friedberg, I.; Hamelryck, T.; Kauff, F.; Wilczynski, B.; De Hoon, M. J. L. Biopython: Freely Available Python Tools for Computational Molecular Biology and Bioinformatics. *Bioinformatics* **2009**, 25 (11), 1422-1423- https://doi.org/10.1093/bioinformatics/btp163.
73. Rousseeuw, P. J. Silhouettes: A Graphical Aid to the Interpretation and Validation of Cluster Analysis. *J. Comput. Appl. Math.* **1987**, 20, 53-65. https://doi.org/10.1016/0377-0427(87)90125-7.
